# Supplementary material for: A multicolor and ratiometric fluorescent sensing platform for metal ions based on arene–metal-ion contact
Source: Commun Chem. 2021 Jul 6;4:104. doi: 10.1038/s42004-021-00541-y (PMC9814090; doi:10.1038/s42004-021-00541-y)
Supplement: Supplementary file 2 — Supplementary information. [file 42004_2021_541_MOESM2_ESM.pdf]

Supplementary Information for

A Multicolor and Ratiometric Fluorescent Sensing Platform for Metal Ions Based on Arene–Metal-Ion Contact

Anna Kanegae, Yusuke Takata, Ippei Takashima, Shohei Uchinomiya, Ryosuke Kawagoe, Akira Yamashita, Kazuteru Usui, Jirarut Wongkongkatep, Manabu Sugimoto, Akio Ojida*

Supplementary Table 1. Summary of the fluorescence emission shifts (nm) of Type-II probes **2**–**4** upon addition of metal ions.[a, b]

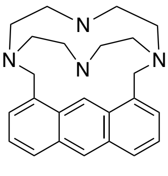

2

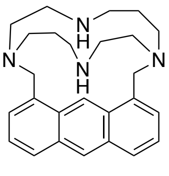

3

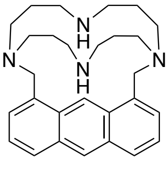

4

	Cr(III)	Mn(II)	Co(II)	Ni(II)	Cu(II)	Zn(II)	Ag(I)	Cd(II)	Pb(II)
**2**	0 (0.73)	0 (1.0)	0 (1.0)	0 (1.0)	0 (0.73)	0 (1.0)	0 (0.96)	0 (1.0)	0 (0.90)[d]
**3**	0 (0.86)	0 (1.04)	0 (1.0)	0 (1.0)	1 (0.01)[c]	0 (0.96)	0 (1.06)	0 (1.0)	0 (0.89)
**4**	0 (0.73)	0 (1.0)	0 (1.0)	0 (1.0)	0 (0.25)	0 (0.96)	0 (0.72)	0 (1.0)	0 (0.93)[d]

[a]Value in the parenthesis indicates fluorescence intensity ratio (F_{max} / F_{o}), where F_{o} and F_{max} designate fluorescence intensity in the absence and presence the maximum concentration of metal ions, respectively. [b]measurement conditions: [probe] = 25 μM, 50 mM HEPES (pH 7.4) / MeOH = 1 : 1, Unless otherwise noted, maximum concentration of metal ion is 1 mM. λ_{ex} = 365 nm, 25 °C. [c]The maximum concentration of metal ion is 100 μM. [d]The maximum concentration of metal ion is 50 μM.

Supplementary Table 2. Summary of the binding constants (K_a, M^{-1}) of probes **5** and **8–11** toward Zn(II) and Cd(II) ion determined by the fluorescence titration (Figure 2, 4, and Supplementary Figure 3).[a,b]

	**5**	**8**	**9**	**10**	**11**
Zn(II)	2.70 x 10^6	5.21 x 10^5	6.49 x 10^5	4.96 x 10^4	1.30 x 10^6
Cd(II)	9.50 x 10^6	1.80 x 10^6	3.73 x 10^6	7.79 x 10^6	2.86 x 10^6

[a]Each value was the average of the two independent experiments. [b]Measurement conditions: [probe] = 1 μM, 50 mM HEPES (pH 7.4) / MeOH = 1 : 1, 25 °C.

Supplementary Table 3. Summary of absorption shifts of probes **5** and **8–11** upon addition of various metal ions.

probe	Cr^{3+}	Mn^{2+}	Co^{2+}	Ni^{2+} (1.63 Å)	Cu^{2+} (1.40 Å)	Zn^{2+} (1.39 Å)	Ag^{+} (1.72 Å)	Cd^{2+} (1.62 Å)	Hg^{2+} (1.70 Å)	Pb^{2+} (2.02 Å)
**5**	-[a]	-[a]	15	6	6	6	6	5	8	11
**8**	-[a]	-[a]	5	<3	<3	6	<3	4	5	-[a]
**9**	-[a]	-[a]	<3	<3	17	10	7	16	16	-[a]
**10**	-[a]	-[a]	<3	<3	24	8	8	20	22	-[a]
**11**	-[a]	-[a]	11	10	12	9	5	14	15	<3

[a]Spectral change was scarcely observed.

Supplementary Table 4. Crystal data of the solved structures of the metal complexes.

	**5**-Zn(II)	**5**-Cu(II)	**5**-Cd(II)	**5**-Ag(I)
experimental formula	$C_{32}H_{33}N_4O_8Cl_2Zn$	$C_{32}H_{33}N_4O_8Cl_2Cu$	$C_{32}H_{33}N_4O_8Cl_2Cd$	$C_{32}H_{33}N_4O_4CAg$
formula weight	750.91	750.09	798.96	693.95
counter anion	ClO_4^-	ClO_4^-	ClO_4^-	ClO_4^-
crystal system	orthorhombic	orthorhombic	monoclinic	monoclinic
a / Å	11.704 (2)	11.651 (19)	41.410 (5)	12.144 (15)
b / Å	16.022 (3)	16.126 (3)	10.669 (13)	8.964 (11)
c / Å	33.430 (6)	33.091 (5)	15.028 (18)	26.531 (3)
α / °	90	90	90	90
β / °	90	90	107.128 (14)	95.491 (10)
γ / °	90	90	90	90
V / Å	6269 (2)	6217.3 (18)	6345.2 (13)	2874 (6)
μ (cm^{-1})	1.013	0.937	0.919	0.84
Z	8	8	8	4
Cristal size / nm	0.2×0.2×0.2	0.2×0.3×0.2	0.3×0.2×0.05	0.2×0.2×0.2
D_{calc} / gcm^{-3}	1.561	1.603	1.673	1.608
F_{000}	3040	3096	3248	1428
radiation	MoKα	MoKα	MoKα	MoKα
T / K	90	90	90	90
No. reflections measured	33682	34255	18178	16444
No. unique reflections	6835	6849	6912	6560
No. reflections observed	7328	7567	7540	6855
No. parameters	465	437	469	420
R_1 (I>2.σ(I))[a]	0.0307	0.0455	0.0361	0.0258
wR_2 (all data)[b]	0.0357	0.1228	0.0633	0.0279
GOF	1.162	1.032	0.989	1.087

[a] $R_1 = \Sigma||F_o|-|F_c|| / \Sigma|F_o|$, [b] $wR_2 = \{\Sigma w(F_o^2-F_c^2)^2 / \Sigma w(F_o^2)^2\}^{1/2}$

Supplementary Table 5. Summary of selected distances and bend angles of the metal ion complexes of **5**.

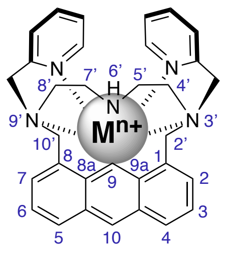

5-M^{n+}

	distance (Å)					bend angle
	C9-metal ion[a]	C9-N6′	N3′-N6′	N6′-N9′	N3′-N9′	C10-C9a-C8a-C9
**5**-Zn(II)	2.96 (1.39 + 1.77)	4.28	2.88	2.81	4.22	+4.9°
**5**-Cd(II)	3.01 (1.58 + 1.77)	4.24	2.99	2.95	4.54	-0.4°
**5**-Ag(I)	3.28 (1.72 + 1.77)	4.51	3.03	2.98	4.43	-0.1°
**5**-Cu(II)	3.14 (1.40 + 1.77)	4.48	2.95	2.80	4.22	+1.7°

[a]The values in the parentheses are the van der Waals radii of each metal ion and aromatic carbon. (1.77 Å).

Supplementary Table 6. Summary of HOMO-LUMO energy gap levels and shape of molecular orbitals of the probes and their Zn(II) complexes.

environment	species	ΔE (ev)	wavelength (nm)	*f*	occupied MO		unoccupied MO	coefficient
PCM (water)	**9**	2.978	416.3	0.8078	138	→	139	0.697
	**9**+Zn(II)	2.733	453.6	0.6177	147	→	148	0.698
PCM (water)	**5**	3.596	344.8	0.1630	130	→	131	0.698
	**5**+Zn(II)	3.520	352.2	0.1523	139	→	140	0.699

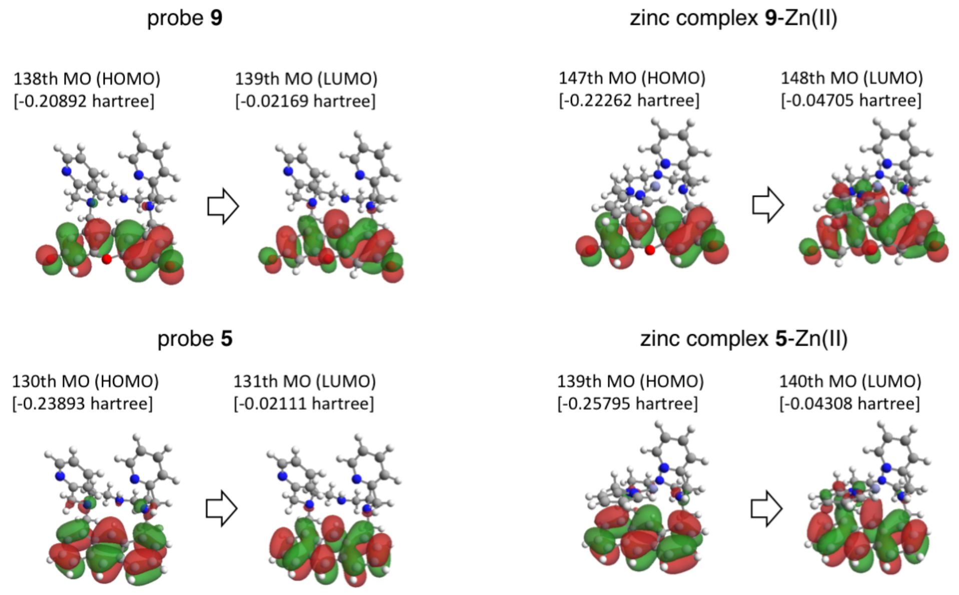

Supplementary Table 7. Summary of S_0-S_1 excitation of the probes with or without coordinated metal ion.

species	optimized geometry[a]	environment	S_0-S_1 excitation		
			ΔE (eV)	l (nm)	*f*
**9**+PC(0.0)	**9**-Zn(II)	vacuum	3.003	412.9	0.570
**9**+Na(I)	**9**-Zn(II)	vacuum	2.812	441.0	0.494
**9**+Ca(II)	**9**-Zn(II)	vacuum	2.451	505.8	0.139
**5**+PC(0.0)	**5**-Zn(II)	vacuum	3.549	349.3	0.113
**5**+Na(I)	**5**-Zn(II)	vacuum	3.576	346.7	0.114
**5**+Ca(II)	**5**-Zn(II)	vacuum	3.555	348.8	0.112

a) Optimized geometry was calculated under vacuum conditions.

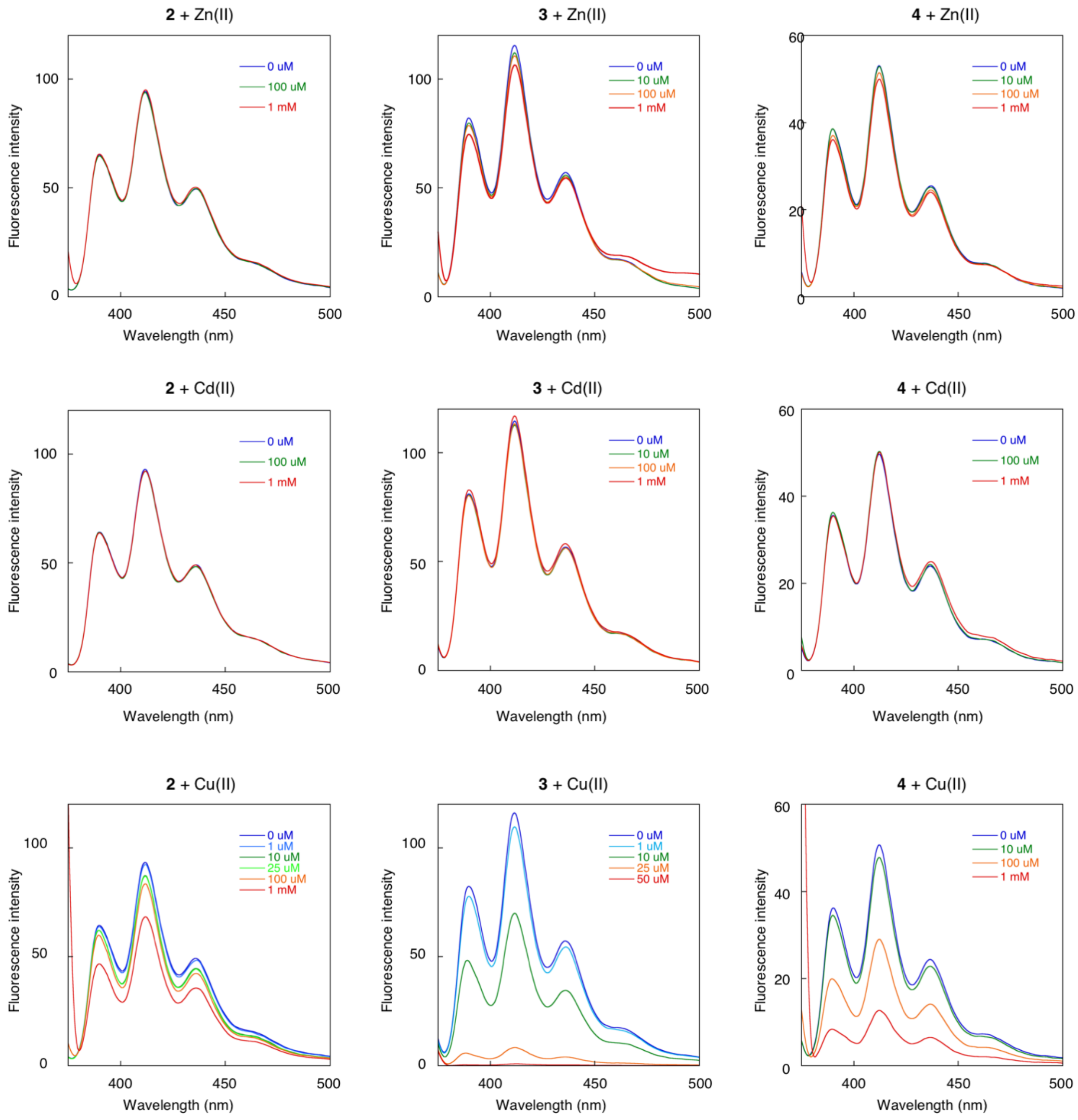

Supplementary Figure 1. Selected results of the fluorescence titration of Type-II probes (see Figure 1, Supplementary Table 1) **2**, **3**, and **4** with metal ions. Measurement conditions: [probe] = 25 μM, 50 mM HEPES (pH 7.4) / MeOH = 1 : 1, λ_{ex} = 365 nm, 25 °C. Counter anion of metal ions is chloride in all the experiments.

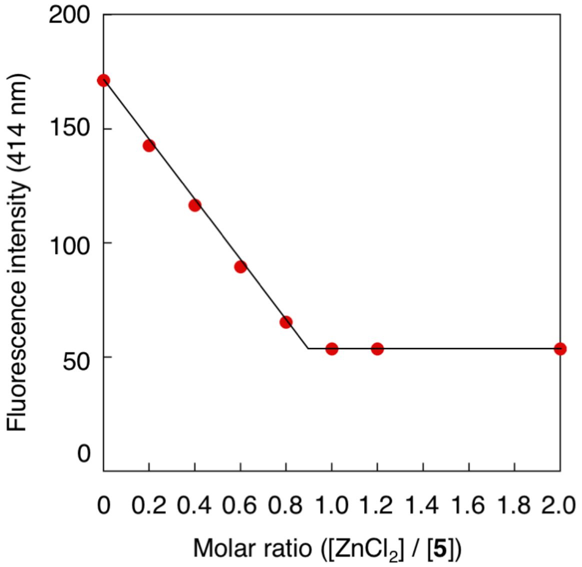

Supplementary Figure 2. Plot of the fluorescence intensity (λ_{em} = 414 nm) of **5** upon addition of $ZnCl_2$. measurement conditions: [**5**] = 25 μM, 50 mM HEPES (pH 7.4) / MeOH = 1 : 1, λ_{ex} = 365 nm, 25 °C.

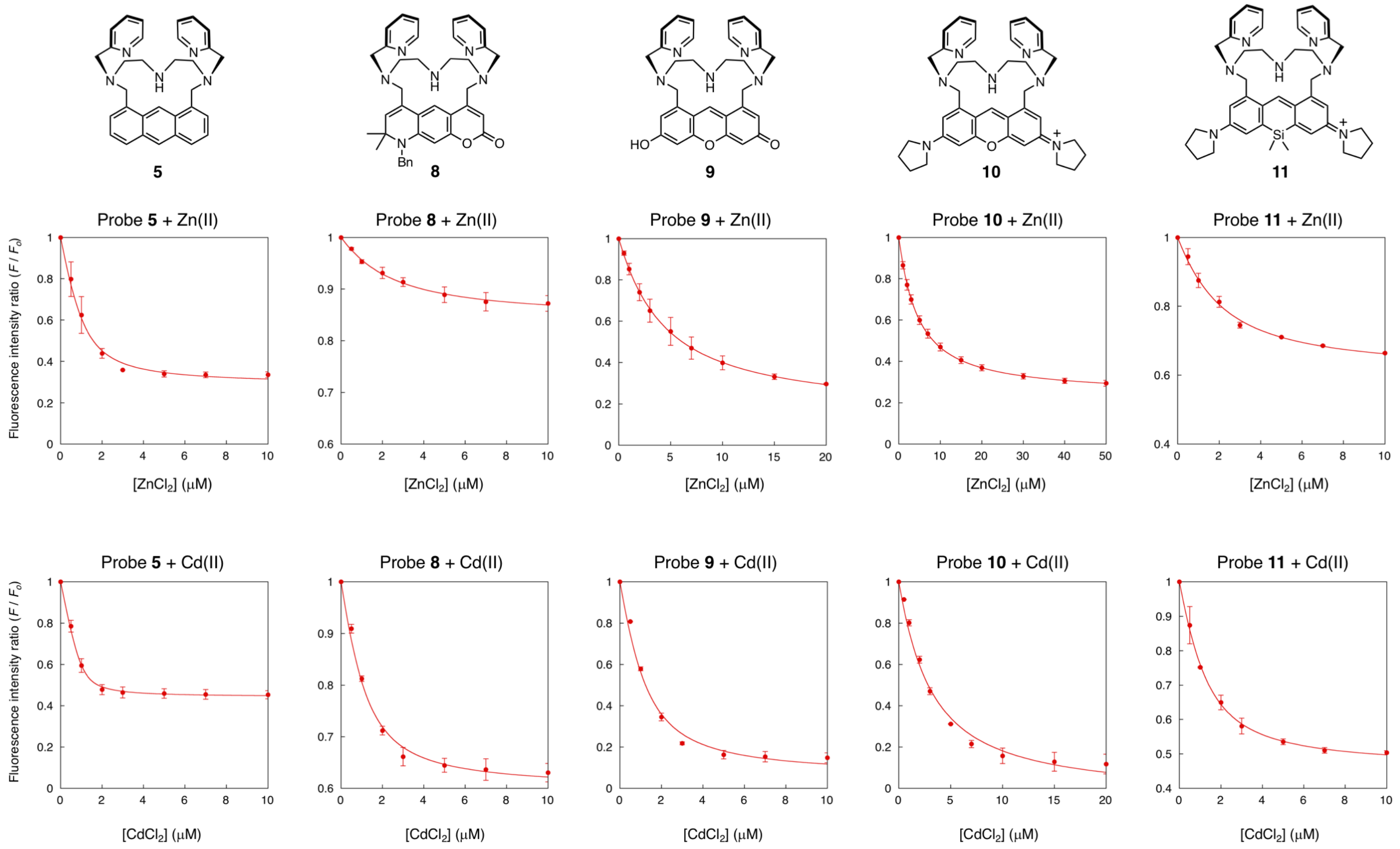

Supplementary Figure 3. Summary of the fluorescence titration profile of the probes with Zn(II) and Cd(II). Measurement conditions: [probe] = 1 μ M, 50 mM HEPES (pH 7.4) / MeOH = 1 : 1, 25 °C. The fluorescence spectrum change of the probes is shown in Figure 2 and Figure 4. The data represent mean ± s.e. of three independent experiments.

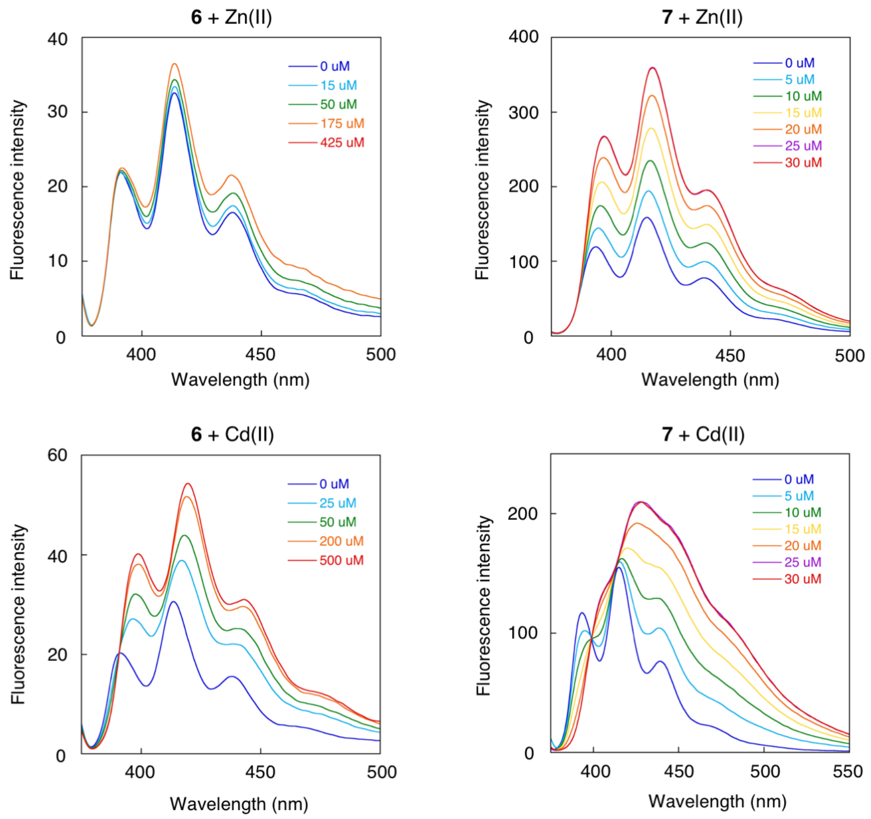

Supplementary Figure 4. Fluorescence spectral changes of probes **6** and **7** upon addition of $ZnCl_2$ or $CdCl_2$. Measurement conditions: [**6** or **7**] = 25 μM, 50 mM HEPES (pH 7.4) / MeOH = 1 : 1, λ_{ex} = 365 nm, 25 °C.

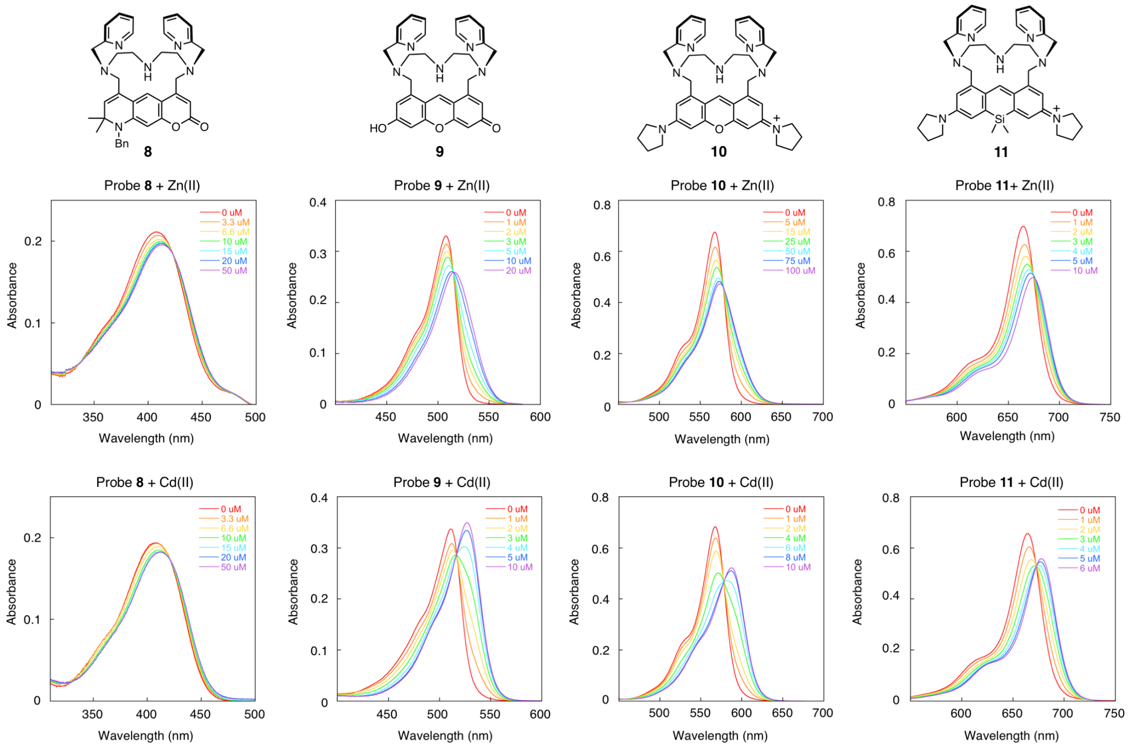

Supplementary Figure 5. UV absorption changes of probes **8**, **9**, **10**, and **11** upon addition of $ZnCl_2$ or $CdCl_2$. Measurement conditions: [probe] = 5 μM (**9**, **10**, **11**) or 10 μM (**8**), 50 mM HEPES (pH 7.4) / MeOH = 1 : 1, 25 °C.

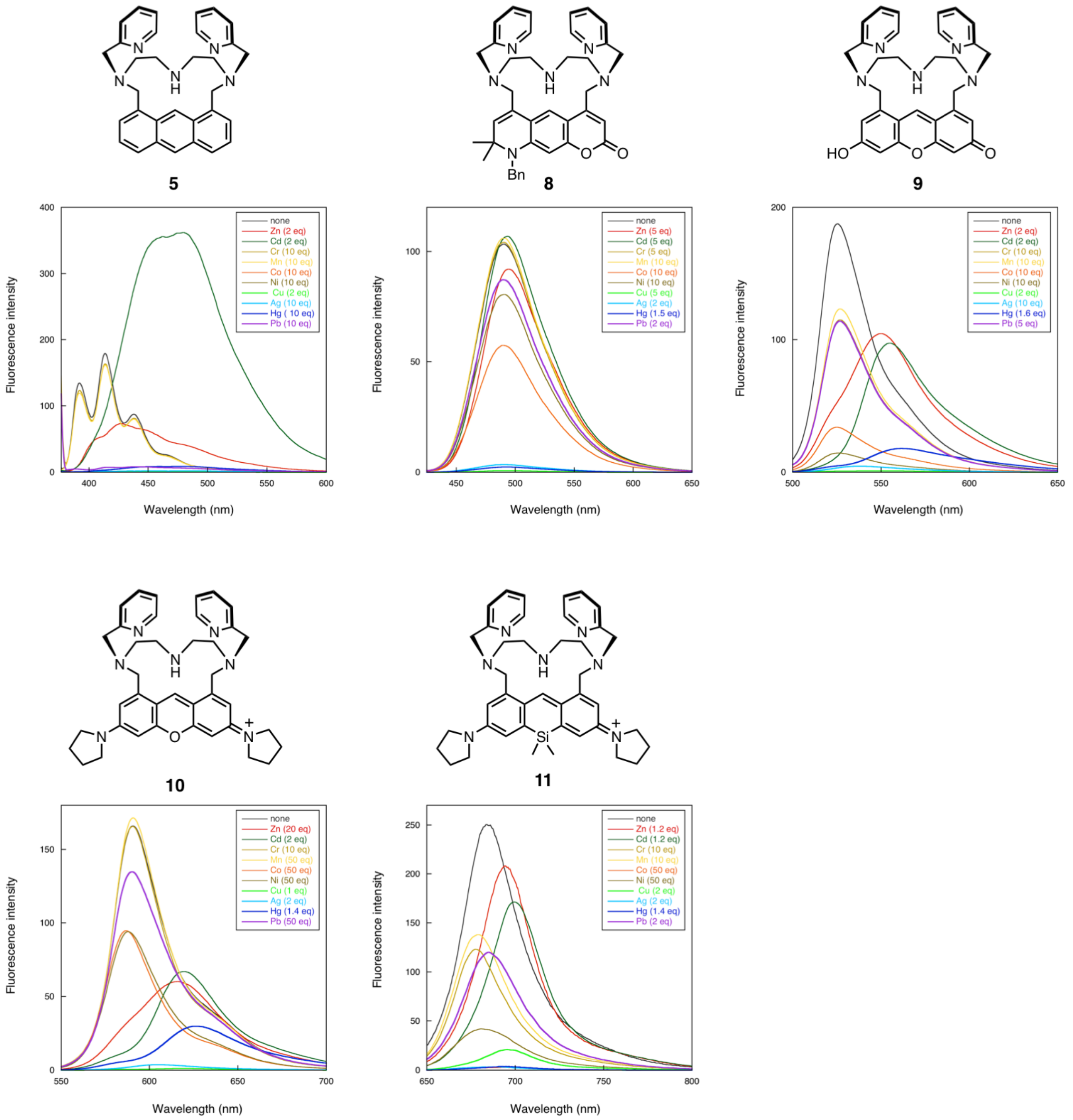

Supplementary Figure 6. Summary of fluorescence spectral changes of probes **5** and **8–11** upon addition of various metal ions. Measurement conditions: [probe] = 25 µM (**5**), 10 µM (**8**), or 5 µM (**9**, **10**, **11**) or, 50 mM HEPES (pH 7.4) / MeOH = 1 : 1, 25 °C. Each spectrum was obtained at the saturation point of the metal ion titration.

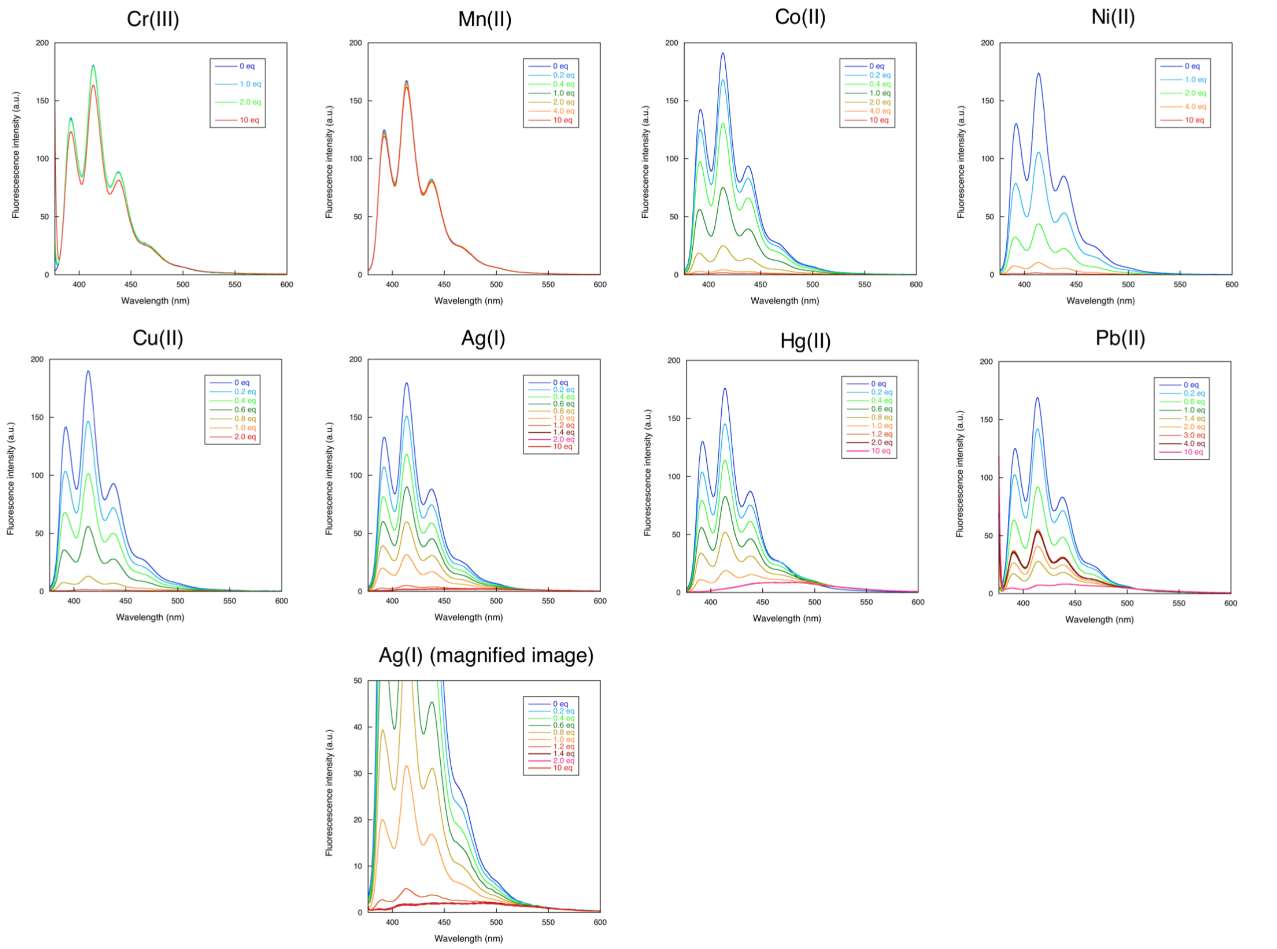

Supplementary Figure 7. Fluorescence spectral change of probe **5** with metal ions. Measurement conditions: [probe] = 25 μ M, 50 mM HEPES (pH 7.4) / MeOH = 1 : 1, 25 °C, λ_{ex} = 365 nm.

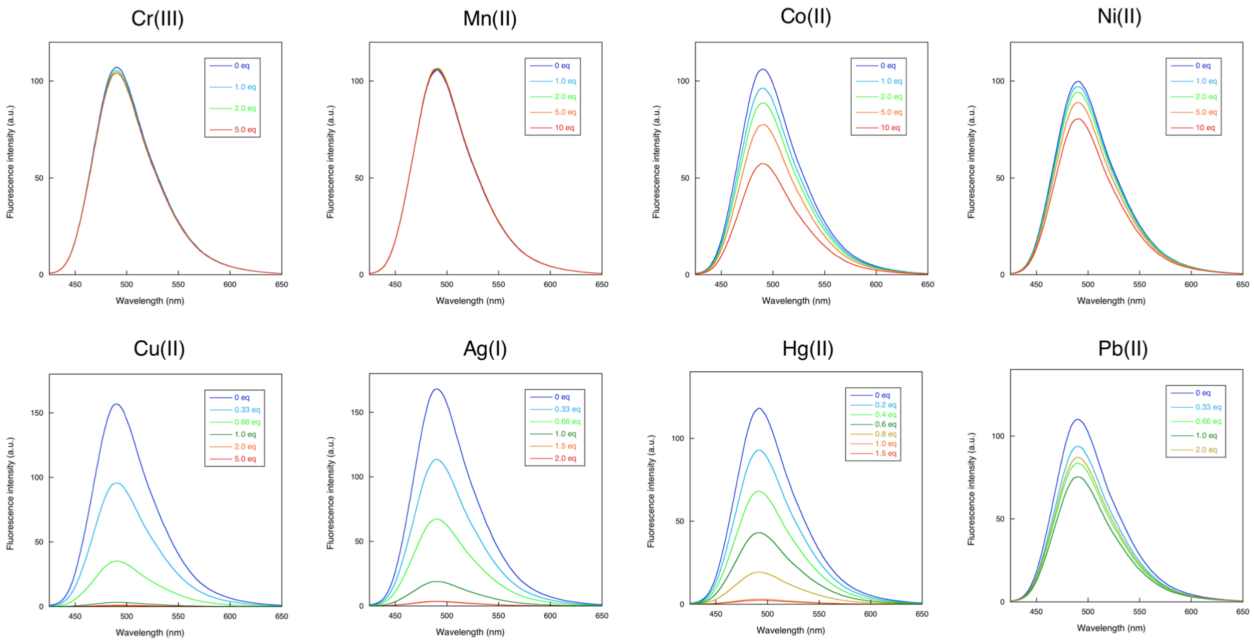

Supplementary Figure 8. Fluorescence spectral change of probe **8** with metal ions. Measurement conditions: [probe] = 10 μ M, 50 mM HEPES (pH 7.4) / MeOH = 1 : 1, 25 °C, λ_{ex} = 410 nm.

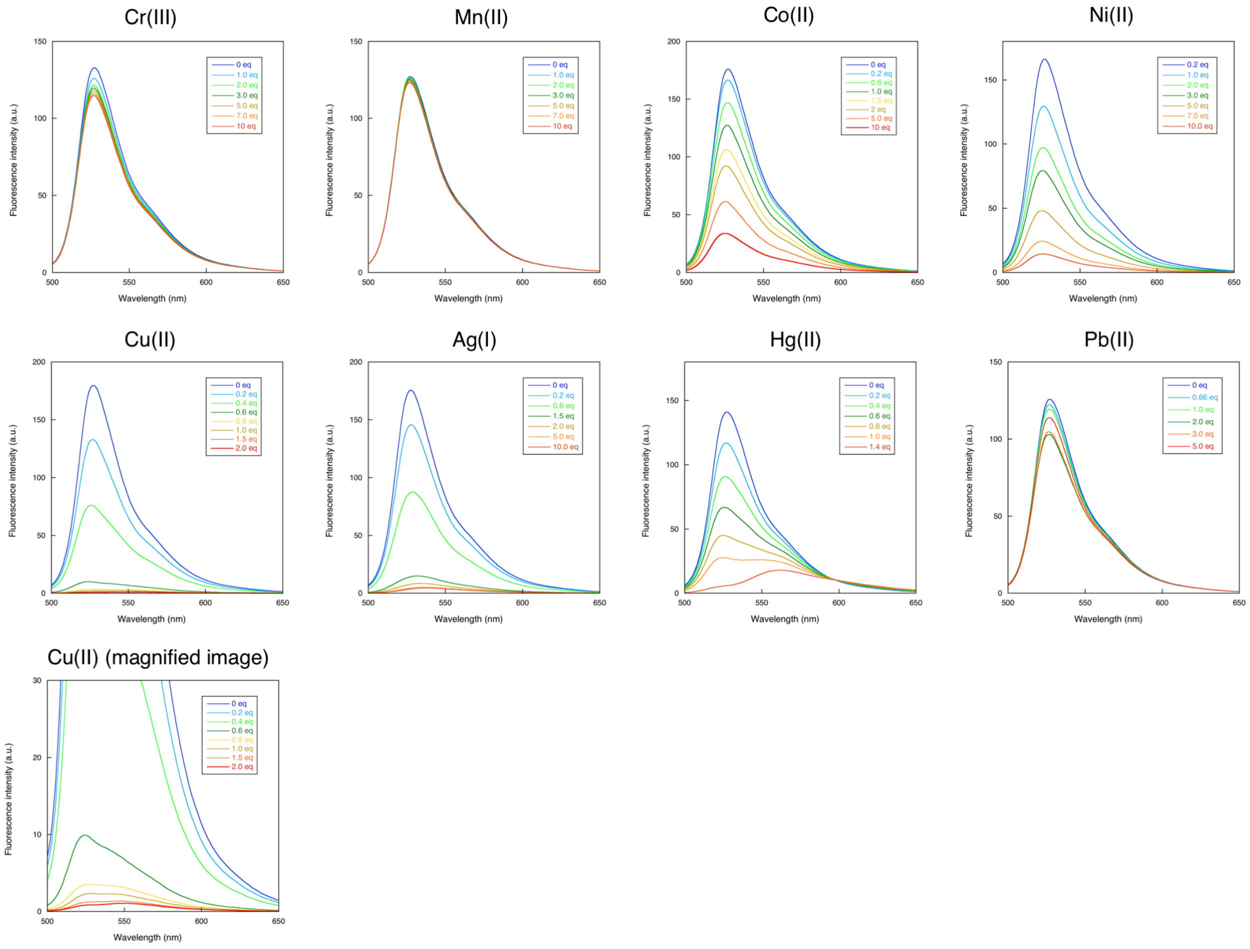

Supplementary Figure 9. Fluorescence spectral change of probe **9** with metal ions. Measurement conditions: [probe] = 5 μ M, 50 mM HEPES (pH 7.4) / MeOH = 1 : 1, 25 °C, λ_{ex} = 488 nm.

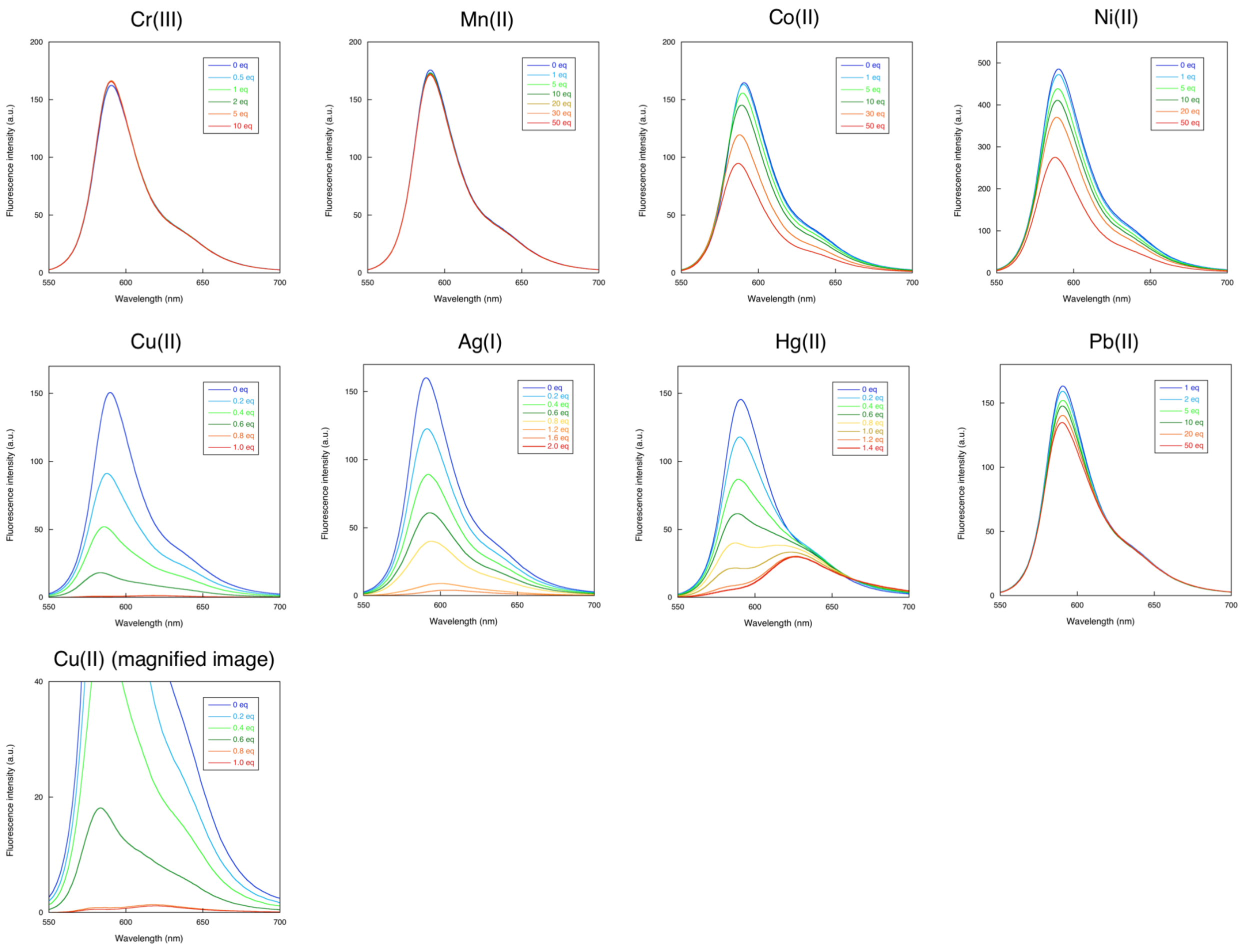

Supplementary Figure 10. Fluorescence spectral change of probe **10** with metal ions. Measurement conditions: [probe] = 5 μ M, 50 mM HEPES (pH 7.4) / MeOH = 1 : 1, 25 °C, λ_{ex} = 578 nm.

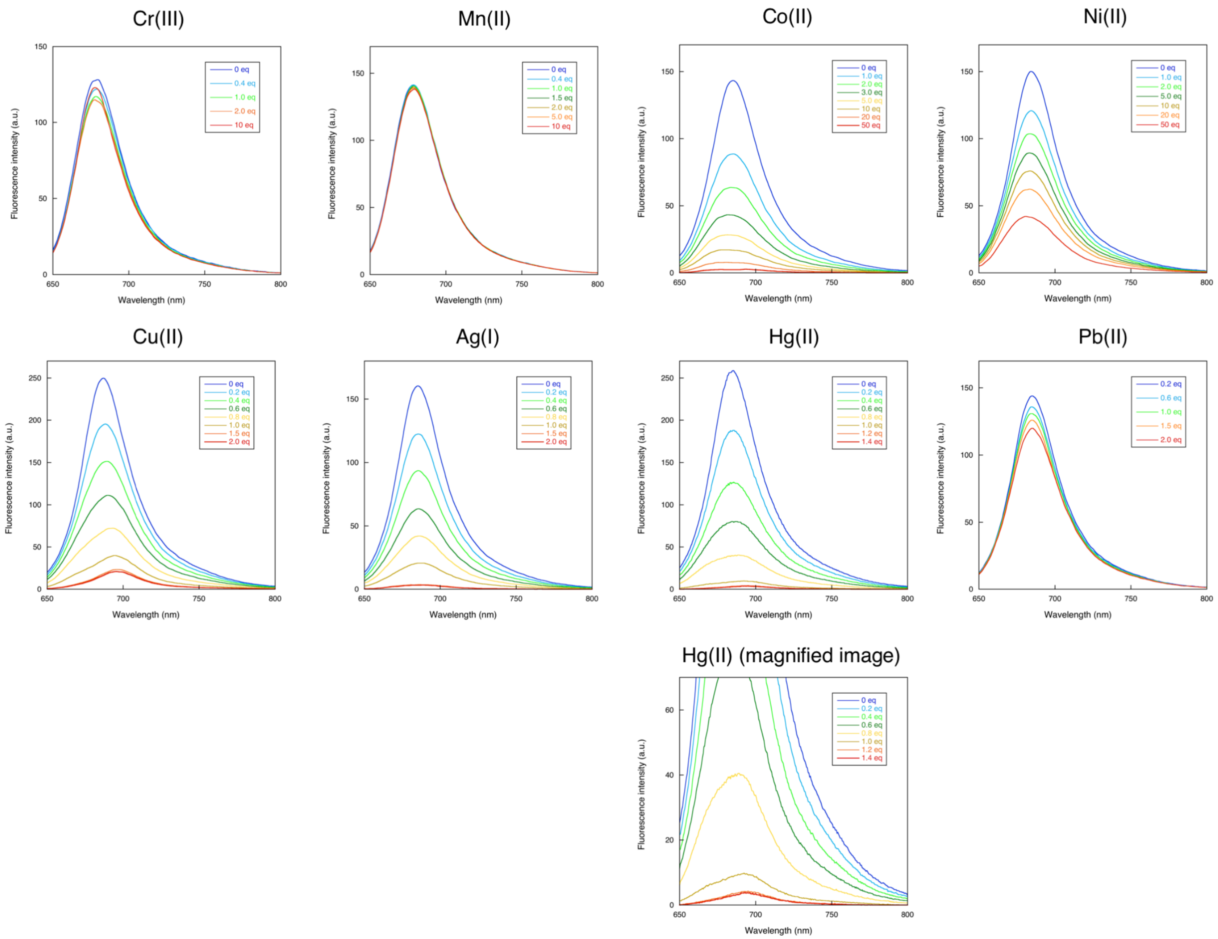

Supplementary Figure 11. Fluorescence spectral change of probe **11** with metal ions. Measurement conditions: [probe] = 5 μ M, 50 mM HEPES (pH 7.4) / MeOH = 1 : 1, 25 °C, λ_{ex} = 674 nm.

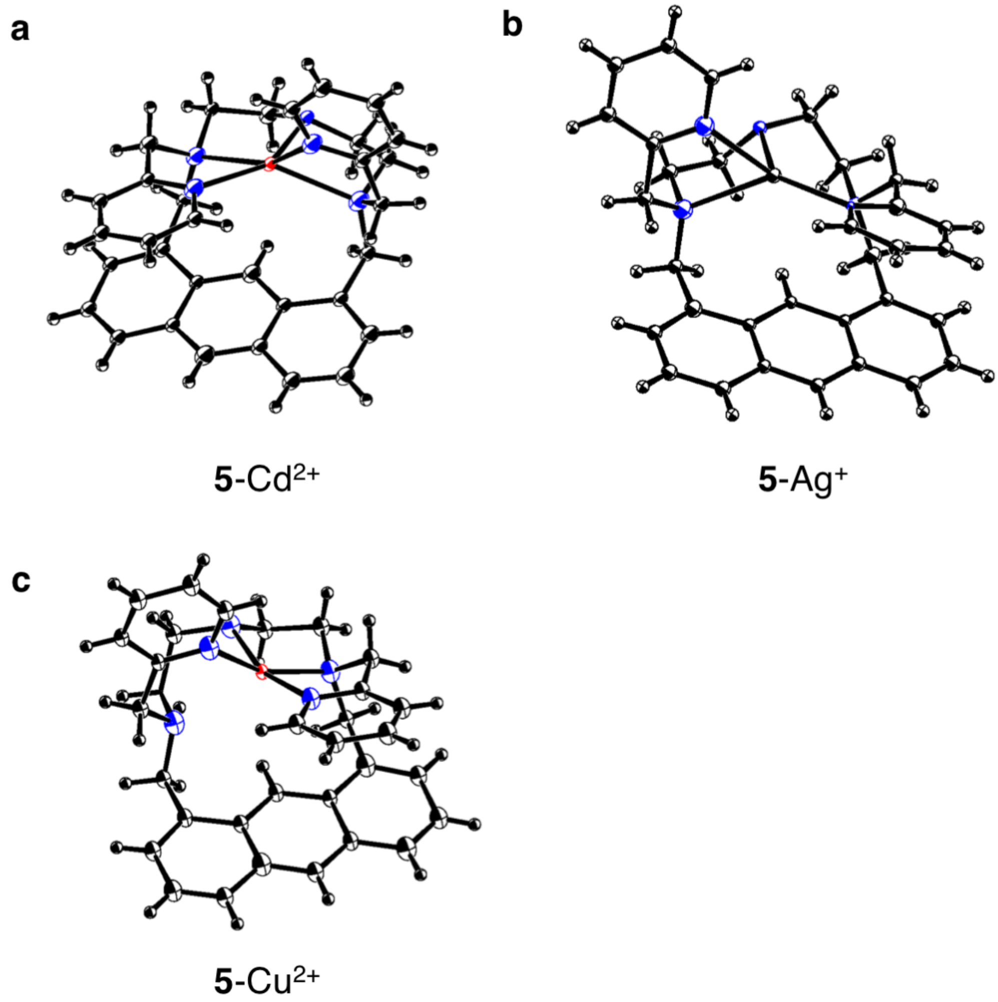

Supplementary Figure 12. ORTEP diagrams (50% probability ellipsoids) of (a) **5**-Cd^{2+} ($C_{32}H_{32}N_4O_8Cl_2Cd$), (b) **5**-Ag^{+} ($C_{32}H_{32}N_4O_4ClAg$), and **5**-Cu^{2+} ($C_{32}H_{32}N_4O_8Cl_2Cu$). The perchlorate anions are omitted for clarity.

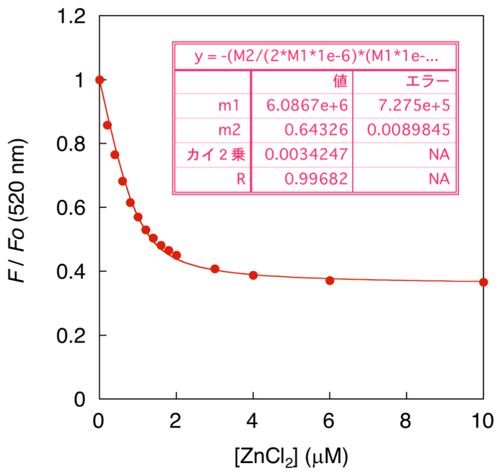

Supplementary Figure 13. Evaluation of the binding constant of probe **12** with Zn(II) under the neutral aqueous conditions: [**12**] = 1 μM, 50 mM HEPES 100 mM NaCl, pH 7.4, 25 °C, λ_{ex} = 488 nm.

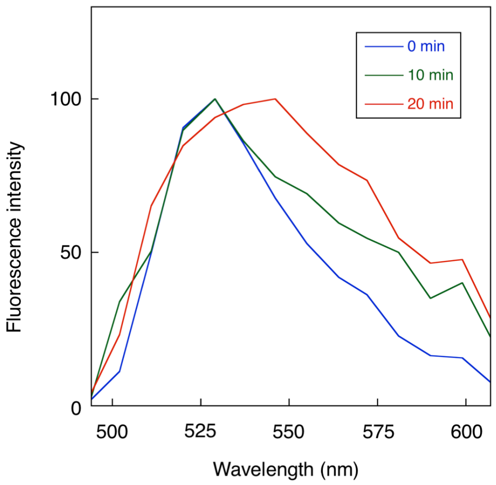

Supplementary Figure 14. Fluorescence change of HeLa cells upon addition of Zn(II) detected by spectral scan mode. The cells were incubated with probe **12** (5 μM) followed by the treatment with $ZnCl_2$ (5 μM) in the presence of pyrithione (100 μM). The fluorescence intensities were measured at the different wavelengths from 499 to 607 nm with 8.8-nm interval.

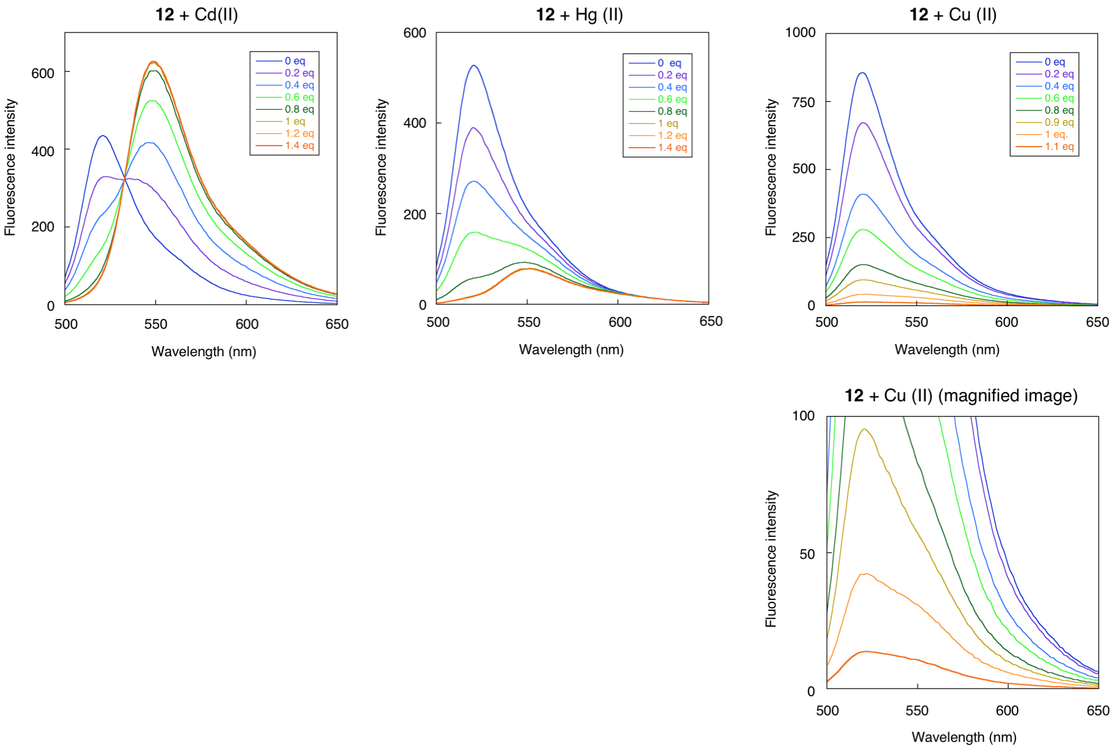

Supplementary Figure 15. Fluorescence spectral change of probe **12** upon addition of Cd(II), Hg(II), and Cu(II) under the neutral aqueous conditions: [**12**] = 1 μM, 50 mM HEPES 100 mM NaCl, pH 7.4, 25 °C, λ_{ex} = 488 nm.

Supplementary Methods

General materials and methods for organic synthesis

Unless otherwise noted, chemical reagents were purchased from commercial suppliers (FUJIFILM Wako Pure Chemical Corporation, Tokyo Chemical Industry, Sigma-Aldrich, Watanabe Chemical Industries) and used without further purification. Reactions were carried out under a positive atmosphere of nitrogen, unless otherwise stated. Reactions were monitored by thin layer chromatography (TLC) carried out on Merck TLC Silica gel 60 F_{254}. ^{1}H-NMR spectra were recorded using a Varian UNITY-400 (400 MHz) spectrometer or Bruker Avance III HD 500 MHz spectrometer and chemical shifts (δ, ppm) were referenced to residual solvent peak ($CDCl_3$: 7.26 ppm; MeOH-d_4: 3.31 ppm; DMSO-d_6: 2.50 ppm). ESI mass spectrometry was recorded using a MicroTOF II (Bruker Daltonics) spectrometer. HPLC purification was conducted with a HITACHI L-7000 (Hitachi).

Synthesis of probe **2**

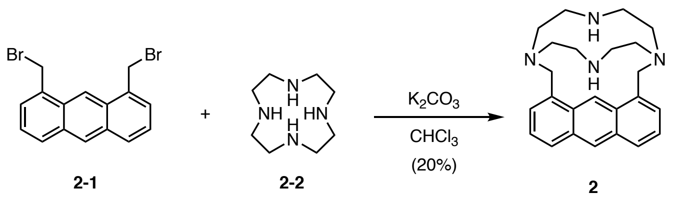

A solution of **2-1**[S1] (25 mg, 68.0 μmol), **2-2** (12 mg, 69.0 μmol) and K_2CO_3 (40 mg, 0.29 mmol) in dry $CHCl_3$ (10 mL) was stirred for 4 h at rt. After dilution with $CHCl_3$, the organic layer was washed with sat. $NaHCO_3$ aq. and brine followed by drying over Na_2SO_4. After removal of the solvent in vacuo, the residue was purified by column chromatography on SiO_2 ($CHCl_3$: MeOH : NH_3 aq. = 20 : 1 : 0.1) to give **2** (10 mg, 20%) as a colorless solid. ^{1}H-NMR (500 MHz, $CDCl_3$): δ 2.67 (16H, brs), 4.26-4.41 (4H, brs), 7.36-7.42 (4H, m), 7.95-7.97 (2H, d, J = 8.5 Hz), 8.47 (1H, s), 9.50 (1H, s). ESI-TOF-MS *m/z* calcd for $C_{24}H_{31}N_4$ $[M+H]^+$ = 375.2549, observed 375.2560.

Synthesis of probe **3**

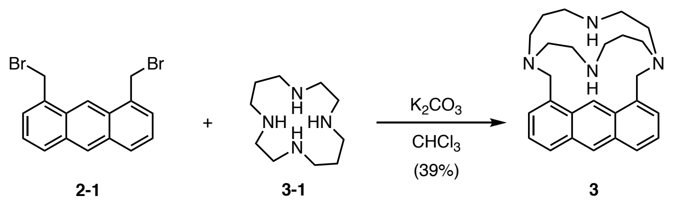

A solution of **2-1** (30 mg, 80.0 μmol), **3-1** (31 mg, 0.12 mmol) and K_2CO_3 (68 mg, 0.50 mmol) in dry $CHCl_3$ (10 mL) was stirred for 7 h at rt. After dilution with $CHCl_3$, the organic layer was

washed with sat. $NaHCO_3$ aq. and brine followed by drying over Na_2SO_4. After removal of the solvent in vacuo, the residue was purified by column chromatography on SiO_2 ($CHCl_3$: MeOH : NH_3 aq. = 200 : 10 : 1) to give **3** (13 mg, 39%) as a colorless solid. ^{1}H-NMR (500 MHz, CD_3OD): δ 1.76 (4H, brs), 2.43-2.46 (4H, m), 2.52 (4H, brs), 2.65 (8H, brs), 3.35 (4H, s), 7.42-7.47 (4H, m), 8.03-8.05 (2H, d, *J* = 8.5 Hz), 8.57 (1H, s), 9.29 (1H, s). ESI-TOF-MS *m/z* calcd for $C_{26}H_{35}N_4$ $[M+H]^+$ = 403.2862, observed 403.2862.

Synthesis of probe **4**

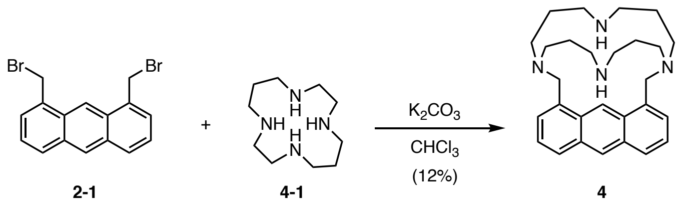

A solution of **2-1** (12 mg, 30 μmol), **4-1**[S2] (14 mg, 30 μmol) and K_2CO_3 (29 mg, 0.21 mmol) in dry $CHCl_3$ (15 mL) was stirred for 2 h at 0 °C. After removal of K_2CO_3 by filtration, the solvent was evaporated. The residue was purified by reverse-phase HPLC (YMC-Triart C18, 250×10 mm I.D., mobile phase gradient: CH_3CN (0.1% TFA) / H_2O (0.1% TFA) = 5/95 → 60/40, linear gradient over 30 min) to give **4** (1.6 mg, 12%) as a colorless solid. ^{1}H-NMR (500 MHz, $CDCl_3$): δ 2.15-2.28 (4H, m), 2.36 (2H, s), 2.57-2.58 (4H, d, *J* = 7.5 Hz), 2.66-2.71 (4H, m), 3.19-3.21 (2H, m), 3.34-3.36 (2H, d, *J* = 12 Hz), 3.42-3.45 (4H, m), 3.49 (4H, s), 4.86-4.89 (2H, d, *J* = 12 Hz), 7.38-7.39 (2H, d, *J* = 6.5 Hz), 7.45-7.48 (2H, m), 8.03-8.04 (2H, d, *J* = 8.5 Hz), 8.55 (1H, s), 8.99 (1H, s). ESI-TOF-MS *m/z* calcd for $C_{28}H_{39}N_4$ $[M+H]^+$ = 431.3175, observed 431.3199.

Synthetic procedure of probe **5**

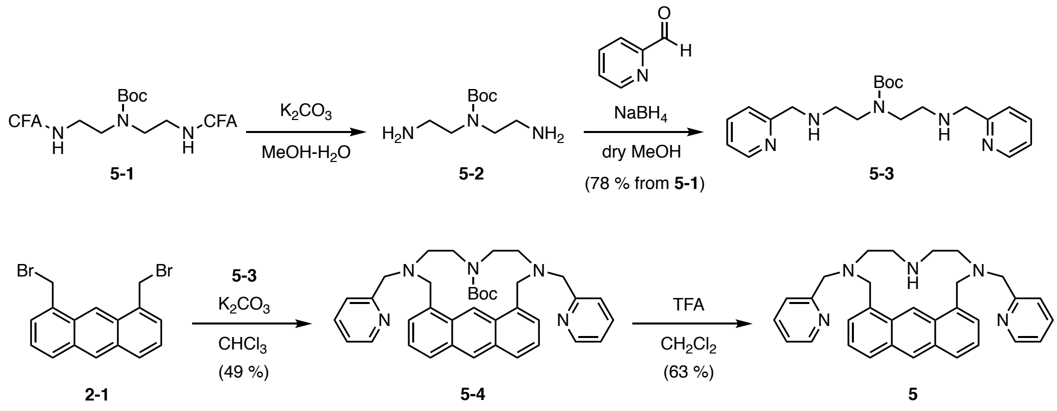

Synthesis of **5-3**

A solution of **5-1**[S3] (2.20 g, 3,81 mmol) and K_2CO_3 (1.80 g, 13.0 mmol) in MeOH (140 mL)-H_2O (7 mL) was heated at 80 °C for 2.5 h with stirring. After removal of the solvent in vacuo, the residue was filtered and washed with EtOH. The filtrate was concentrated by evaporation to give crude **5-2**, which was dissolved in EtOH and concentrated by evaporation twice to azeotropically remove H_2O. To a solution of crude **5-2** in dry MeOH (30 mL) added 2-formylpyridine (898 mg, 8.38 mmol) dissolved in dry MeOH (10 mL), and the mixture was stirred overnight at rt. Sodium borohydride (361 mg, 9.53 mmol) was added portionwise at 0 °C and the mixture was further stirred for 3 h at rt. After removal of the solvent by evaporation, the residue was diluted with sat. $NaHCO_3$ aq. and extracted with $CHCl_3$ (x2). The combined organic layers were dried over Na_2SO_4 and concentrated in vacuo. The residue was purified by column chromatography on SiO_2 ($CHCl_3$: MeOH : NH_3 aq. = 10 : 1 : 0.1 → 8 : 1: 0.1) to give **5-3** (1.15 g, 78%) as a pale yellow viscous oil.^{1}H-NMR (500 MHz, $CDCl_3$): δ 1.42 (9H, s), 2.80-2.83 (4H, t, J = 6.3 Hz), 3.38 (4H, s), 3.90 (4H, s), 7.13-7.15 (2H, m), 7.28-7.30 (2H, d, J = 8.0 Hz), 7.60-7.64 (2H, m), 8.53 (2H, d, J = 4.5 Hz). ^{13}C-NMR (125 MHz, $CDCl_3$): δ 159.3, 155.3, 148.8, 136.0, 121.7, 121.5, 79.1, 77.2, 54.5, 47.5, 28.0. .ESI-TOF-MS *m/z* calcd for $C_{21}H_{32}N_5O_2$ $[M+H]^+$ = 386.2556, observed 386.2498.

Synthesis of **5-4**

To a solution of **5-3** (40.4 mg, 0.10 mmol), **2-1** (38 mg, 0.10 mmol) and triethylamine (29 μL, 21.1 mmol) in dry $CHCl_3$ (15 mL) was stirred at 40 °C for 24 h. After dilution with $CHCl_3$, the organic layer was washed with sat. $NaHCO_3$ aq. and brine followed by drying over Na_2SO_4. After removal of the solvent in vacuo, the residue was purified by column chromatography on SiO_2 ($CHCl_3$: MeOH : NH_3 aq. = 40 : 1 : 0.1) to give **5-3** (28.3 mg, 49%) as a yellow solid. ^{1}H-NMR (500 MHz, $CDCl_3$): δ 1.27 (9H, s), 2.85 (4H, brs), 3.69 (4H, brs), 3.77 (4H, s), 4.23 (4H, brs), 7.02(2H, t, J = 5.5 Hz), 7.14 (2H, d, J = 7.5 Hz), 7.37-7.43 (6H, m), 7.96 (2H, d, J = 8.5 Hz). 8.45 (s, 2H), 10.1 (s, 1H). ESI-TOF-MS *m/z* calcd for $C_{21}H_{32}N_5O_2$ $[M+H]^+$ = 588.3339, observed 588.3501.

Synthesis of **5**

To an ice-cooled solution of **5-4** (28.0 mg, 48 μmol) in dry CH_2Cl_2 (1 mL) was added dropwise TFA (1 mL), and the mixture was stirred for 30 min at rt. After removal of the solvent in vacuo, the residue was dissolved in $CHCl_3$ and washed with sat. $NaHCO_3$ aq. and brine followed by drying over Na_2SO_4. After removal of the solvent in vacuo, the residue was purified by column chromatography on SiO_2 ($CHCl_3$: MeOH : NH_3 aq. = 40 : 1 : 0.1) to give **5** (14.4 mg, 63%) as a pale yellow solid. H-NMR (500 MHz, $CDCl_3$): δ 2.90 (4H, brs), 3.05 (4H, brs), 3.76 (4H, brs), 6.94 (2H, t, J = 6.3 Hz), 7.03 (2H, t, J = 7.0 Hz), 7.19 (2H, td, J = 1.7 Hz,), 7.37 (1H, d, J = 8.4

Hz), 7.38 (1H, d, J = 8.4 Hz), 7.47 (2H, d, J = 6.5 Hz), 7.93 (2H, d, J = 8.5 Hz), 8.37 (2H, d, J = 4.3 Hz), 8.43 (1H, s), 9.38 (1H, brs). ^{13}C-NMR (125 MHz, $CDCl_3$): δ 160.1, 148.5, 136.0, 134.2, 132.0, 131.0, 131.0, 130.1, 128.9, 127.9, 124.6, 122.8, 121.4, 120.3, 60.9, 58.2, 55.9, 46.7. ESI-TOF-MS *m/z* calcd for $C_{32}H_{34}N_5$ $[M+H]^+$ = 488.2814, observed 488.2763.

Synthetic procedure of probe **6**

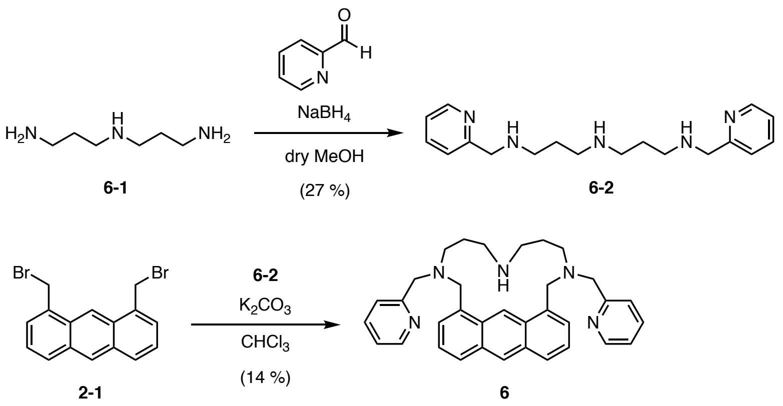

Synthesis of **6-2**

To a solution of **6-1** (1.57 g, 12 mmol) in dry MeOH (35 mL) was added 2-formyl pyridine (2.57 g, 24 mmol) and the mixture was stirred overnight at room temperature. Sodium borohydride (1.30 g, 34.3 mmol) was added and the mixture was further stirred overnight at rt. After removal of the solvent by evaporation, the residue was diluted with water and extracted with $CHCl_3$ followed by drying over Na_2SO_4. The organic layer was concentrated in vacuo and the residue was purified by column chromatography on SiO_2 ($CHCl_3$: MeOH : NH_3 aq. = 10 : 1 : 0.1) to give **6-2** (1.01 g, 27%) as a colorless oil. ^{1}H NMR (500 MHz, $CDCl_3$): δ 1.69-1.75 (4H, m), 2.67-2.73 (8H, m), 3.89 (4H, s), 7.13-7.16 (2H, m), 7.29-7.30 (2H, d, J = 7.5 Hz), 7.61-7.65 (2H, m), 8.54-8.56 (2H, m). ESI-TOF-MS *m/z* calcd for $C_{18}H_{28}N_5$ $[M+H]^+$ = 314.2345, observed 314.2501.

Synthesis of **6**

A solution of **6-2** (50.3 mg, 0.16 mmol), **2-1** (58.4 mg, 0.16 mmol) and K_2CO_3 (44 mg, 0.32 mmol) in dry $CHCl_3$ (15 mL) was stirred for 3 h at rt. After dilution with $CHCl_3$, the organic layer was washed with sat. $NaHCO_3$ aq. and brine followed by drying over Na_2SO_4. After removal of the solvent in vacuo, the residue was purified by column chromatography on SiO_2 ($CHCl_3$: MeOH : NH_3 aq. = 20 : 10 : 0.1) to give **6** (11.5 mg, 14%) as a pale yellow solid. ^{1}H-NMR (500 MHz, $CDCl_3$): δ 2.08 (4H, brs), 2.64 (4H, brs), 2.68-2.71 (4H, t, J = 5.5 Hz), 4.33 (4H, s), 4.38 (4H, brs), 7.16-7.18 (2H, m), 7.34-7.39 (6H, m), 7.60-7.64 (2H, m), 7.92-7.93 (2H, d, J = 8.0 Hz), 8.41 (1H, s), 8.60-8.61 (2H, d, J = 4.5 Hz), 9.27 (1H, s). ESI-TOF-MS *m/z* calcd for $C_{34}H_{38}N_5$

$[M+H]^+$ = 516.3127, observed 516.3343.

Synthesis of **7**

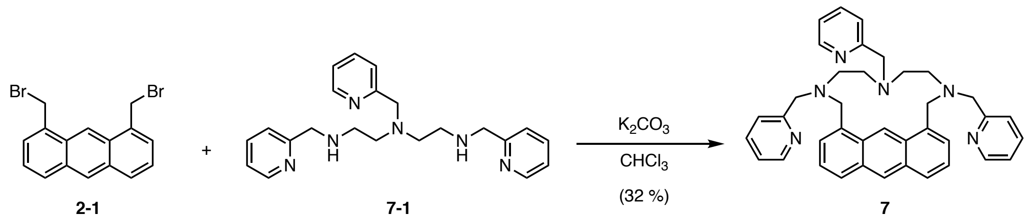

A solution of **7-1**[S4] (29 mg, 0.077 mmol), **2-1** (28 mg, 0.077 mmol) and K_2CO_3 (25 mg, 0.18 mmol) in dry DMF (10 mL) was stirred for 3 h at rt. After dilution with AcOEt, the organic layer was washed with sat. $NaHCO_3$ aq. and brine followed by drying over Na_2SO_4. After removal of the solvent in vacuo, the residue was purified by column chromatography on SiO_2 ($CHCl_3$: MeOH : NH_3 aq. = 20 : 1 : 0.1) to give **7** (14.1 mg, 32%) as a pale yellow solid. ^{1}H-NMR (500 MHz, $CDCl_3$): δ 2.82-2.89 (8H, brs), 3.59-3.69 (6H, brs), 4.34 (4H, brs), 6.90-6.93 (2H, m), 6.99-7.05 (4H, m), 7.14-7.17 (3H, m), 7.37-7.39 (5H, m), 7.94-7.96 (2H, dd, *J* = 2.5, 7.0 Hz), 8.36-8.43 (4H, m). ESI-TOF-MS *m/z* calcd for $C_{38}H_{39}N_6$ $[M+H]^+$ = 579.3236, observed 579.3266.

Synthetic procedure of probe **8**

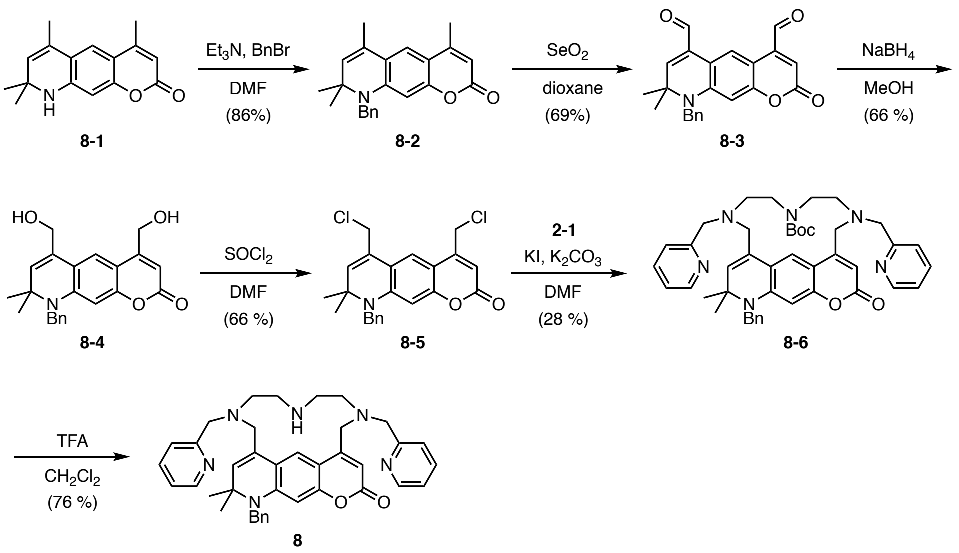

Synthesis of **8-2**

A solution of **8-1**[S5] (660 mg, 2.59 mmol), benzyl bromide (0.93 mL, 7.76 mmol) and triethyl amine (0.54 mL, 3.88 mmol) in dry DMF (20 mL) was stirred for 15 h at 110 °C. After dilution

with sat. $NaHCO_3$ aq., the mixture was extracted with ethyl acetate (x2). The combined organic layers were washed with sat. $NaHCO_3$ aq. and brine followed by drying over Na_2SO_4. After removal of the solvent in vacuo, the residue was purified by column chromatography on SiO_2 (hexane : AcOEt = 1 : 1) to give **8-2** (773 mg, 86%) as a yellow powder. ^{1}H-NMR (400 MHz, $CDCl_3$) : δ 1.42 (6H, s), 2.08 (3H, s), 2.33 (3H, s), 4.56 (1H, s), 5.41 (1H, s), 5.90 (1H, s), 6.12 (1H, s), 7.14 (1H, s), 7.22-7.32 (5H, m). ESI-TOF-MS *m/z* calcd for $C_{23}H_{24}NO_2$ $[M+H]^+$ = 346.1807, observed 346.1810.

Synthesis of **8-3**

A solution of **8-2** (867.2 mg, 2.50 mmol) and SeO_2 (777 mg, 7.50 mmol) in dry dioxane (50 mL) was refluxed for 22 h with stirring. After removal of the solvent in vacuo, the residue was purified by column chromatography on SiO_2 (hexane : AcOEt = 1 : 1) to give **8-3** (621 mg, 69%) as a red powder. ^{1}H-NMR (400 MHz, $CDCl_3$) : δ 1.55 (6H, s), 4.61 (2H, s), 6.28 (1H, s), 6.36 (1H, s), 6.48 (1H, s), 7.24 (1H, s), 7.24-7.35 (5H, m), 9.37 (1H, s), 9.72 (1H, s). ESI-TOF-MS *m/z* calcd for $C_{23}H_{20}NO_4$ $[M+H]^+$ = 374.1392, observed 374.1401.

Synthesis of **8-4**

To a solution of **8-3** (421 mg, 1.13 mmol) in dry MeOH (20 mL) was added portionwise $NaBH_4$ (102 mg, 2.70 mmol) and the mixture was stirred for 12 h at rt. After addition of water, MeOH was removed by evaporation. The aqueous solution was neutralized with 1N HCl and extracted with CH_2Cl_2 (x4). The combined organic layers were dried over $MgSO_4$ and concentrated by evaporation. The residue was purified by column chromatography on SiO_2 ($CHCl_3$: MeOH = 20 : 1) to give **8-4** (279 mg, 66 %) as a yellow powder. ^{1}H-NMR (400 MHz, $CDCl_3$) : δ 1.45 (6H, s), 4.52 (2H, d, J = 5.6 Hz), 4.57 (2H, s), 4.79-4.81 (2H, d, J = 5.6 Hz), 5.66 (1H, s), 6.18 (1H, s), 6.20 (1H, s), 7.23-7.30 (6H, m). ^{13}C-NMR (125 MHz, $CDCl_3$) : δ 162.4, 155.6, 154.7, 147.9, 137.2, 130.7, 129.7, 128.8, 127.1, 125.9, 117.8, 117.2, 106.9, 106.1, 99.3, 63.1, 60.8, 57.9, 48.3, 29.0. ESI-TOF-MS *m/z* calcd for $C_{23}H_{24}NO_4$ $[M+H]^+$ = 378.1705, observed 378.1714.

Synthesis of **8-5**

To a solution of **8-4** (80.5 mg, 0.21 mmol) in dry DMF (4 mL) was added dropwise thionyl chloride (80 μL, 1.10 mmol) at rt. The mixture was stirred for 15 min at rt. After dilution with AcOEt, the organic layer with sat. $NaHCO_3$ aq. and brine followed by drying over Na_2SO_4. After removal of the solvent in vacuo, the residue was purified by column chromatography on SiO_2 (hexane : AcOEt = 2 : 1) to give **8-5** (69 mg, 69%) as a yellow powder. ^{1}H-NMR (400 MHz, $CDCl_3$) : δ 1.47 (6H, s), 4.43 (2H, s), 4.57 (4H, s), 5.75 (1H, s), 6.17 (1H, s), 6.19 (1H, s), 7.24-7.31 (5H, m), 7.42 (1H, s). ^{13}C-NMR (125 MHz, $CDCl_3$) : δ 161.2, 156.0, 149.7, 148.0, 136.7,

133.3, 128.8, 127.5, 127.2, 125.7, 118.9, 116.2, 109.8, 106.8, 99.6, 58.1, 48.3, 44.2, 41.4, 28.8. ESI-TOF-MS *m/z* calcd for $C_{23}H_{22}Cl_2NO_2$ $[M+H]^+$ = 414.1028, observed 414.1036.

Synthesis of **8-6**

A solution of **8-5** (42.2 mg, 0.10 mmol), **5-3** (50 mg, 0.13 mmol), potassium iodide (20 mg, 0.12 mmol), and K_2CO_3 (45 mg, 0.33 mmol) in dry DMF (10 mL) was stirred for 9 h at rt. After dilution with AcOEt, the organic layer was washed with sat. $NaHCO_3$ aq. (x2) and brine followed by drying over Na_2SO_4. The solvent was removed in vacuo and the residue was purified by column chromatography on SiO_2 ($CHCl_3$: MeOH : NH_3 aq. = 30 : 10 : 0.3) to give **8-6** (40 mg, 55%) as an orange solid. ^{1}H-NMR (400 MHz, $CDCl_3$) : δ 1.28 (9H, s), 1.39 (6H, s), 2.75-2.77 (4H, t, *J* = 4.0 Hz), 3.51-3.54 (6H, d, *J* = 13.2 Hz), 3.75-3.78 (8H, m), 4.54 (2H, s), 5.56 (1H, s), 6.17 (1H, s), 6.19 (1H, s), 7.12 (2H, m), 7.24-7.31 (7H, m), 7.55 (3H, t, *J* = 8.8 Hz), 8.50 (2H, t, *J* = 5.2 Hz). ESI-TOF-MS *m/z* calcd for $C_{44}H_{51}N_6O_4$ $[M+H]^+$ = 727.3972, observed 727.3981.

Synthesis of **8**

To an ice-cooled solution of crude **8-6** (14.7 mg, 20 μmol) in dry CH_2Cl_2 (2 mL) was added dropwise TFA (2 mL), and the mixture was stirred for 30 min at rt. After removal of the solvent in vacuo, the mixture was purified by reverse-phase HPLC (YMC-Triart C18, 250×10 mm I.D., mobile phase: CH_3CN (0.1% TFA) / H_2O (0.1% TFA) = 20/80 → 60/40, linear gradient over 40 min) to give **8** (6.8 mg, 54%) as an orange solid. The purity of **9** was confirmed to be > 95% by HPLC analysis. ^{1}H-NMR (500 MHz, DMSO-d_6) : δ = 1.36 (6H, s), 2.60-2.82 (8H, m), 3.42-3.95 (8H, m), 4.64 (2H, s), 5.74 (1H, s), 6.02 (1H, s), 6.09 (1H, s), 7.10-7.15 (1H, m), 7.18-7.29 (5H, m), 7.29-7.39 (3H, m), 7.47 (1H, td, *J* = 7.7 Hz, 1.7 Hz), 7.58 (1H, td, *J* = 7.7 Hz, 1.7 Hz), 7.99 (1H, s), 8.35 (1H, s) 8.41 (1H, dd, *J* = 4.8 Hz, 0.75 Hz), 8.48 (1H, dd, *J* = 4.8 Hz, 0.75 Hz). ^{13}C-NMR (125 MHz, DMSO-d_6) : δ = 160.1, 160.0, 158.8, 155.2, 152.5, 148.5, 148.4, 147.3, 138.2, 136.1, 135.8, 135.2, 128.5, 126.9, 126.6, 125.9, 122.9, 122.5, 121.8, 121.7, 121.2, 117.8, 111.8, 108.0, 98.3, 79.2, 79.0, 78.7, 69.8, 59.7, 59.5, 57.6, 57.3, 55.5, 55.2, 47.5, 45.3, 45.2, 28.4. ESI-TOF-MS *m/z* calcd for $C_{39}H_{42}N_6O_2$ $[M+H]^+$ = 627.3447, observed 627.3506.

Synthetic procedure of probe **9**

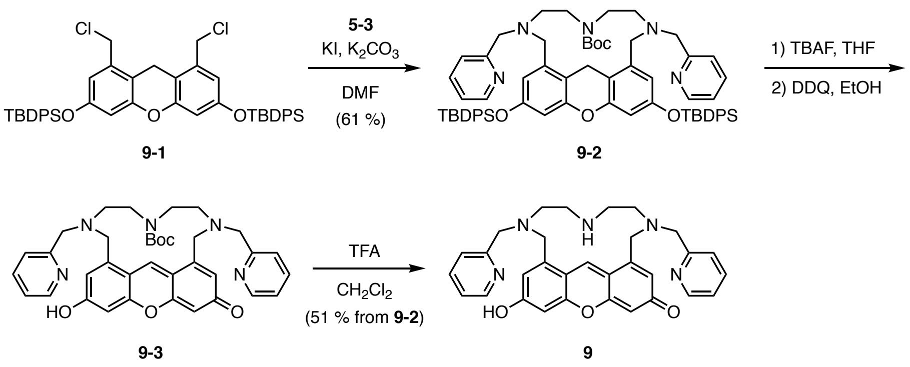

Synthesis of **9-2**

A solution of **9-1**[S6] (60 mg, 76 μmol), **5-3** (42 mg, 0.11 mmol), potassium iodide (13 mg, 78 μmol) and K_2CO_3 (32 mg, 0.23 mmol) in dry DMF (6 mL) was stirred for 5.5 h at rt. After dilution with sat. $NaHCO_3$ aq., the mixture was extracted with AcOEt (x2). The combined organic layers were washed with sat. $NaHCO_3$ aq. and brine followed by drying over Na_2SO_4. After removal of the solvent in vacuo, the residue was purified by column chromatography on SiO_2 ($CHCl_3$: MeOH : NH_3 aq. = 80 : 1 : 0.1) to give **9-2** (51 mg, 61%) as a pale yellow oil. ^{1}H-NMR (500 MHz, $CDCl_3$): δ 1.09 (18H, s), 1.22 (9H, s), 2.52-2.54 (4H, d, J = 5.8 Hz), 3.29 (4H, s), 3.39 (4H, s), 3.42 (4H, s), 4.26 (2H, s), 6.26-6.27 (2H, d, J = 2.5 Hz), 6.45 (2H, d, J = 2.5 Hz), 6.97-6.99 (2H, d, J = 7.5 Hz), 7.05-7.08 (2H, m), 7.29-7.34 (12H, m), 7.41 (2H, s), 7.68-7.70 (8H, m), 8.44-8.45 (2H, d, J = 4.6 Hz). ESI-TOF-MS m/z calcd for $C_{68}H_{78}N_5O_5Si_2$ $[M+H]^+$ = 1100.5541, observed 1100.4864.

Synthesis of **9-3**

To a solution of **9-2** (51 mg, 46 μmol) in dry THF (2 mL) was added tetrabutylammonium fluoride in THF (1 M; 200 μL, 200 μmol), and the mixture was stirred for 1 h at rt. After removal of the solvent in vacuo, the residue was dissolved in dry EtOH (3 mL). 2,3-Dichloro-5,6-dicyano-*p*-benzoquinone (11 mg, 48 μmol) was added and the mixture was stirred for 15 min at rt. After removal of the solvent in vacuo, the residue was purified by column chromatography on SiO_2 ($CHCl_3$: MeOH : NH_3 aq. = 10 : 1 : 0.1) to give crude **9-3** (35 mg) as an orange oil. ^{1}H-NMR (500 MHz, $CDCl_3$): 1.27 (9H, s), 2.72 (4H, s), 3.53 (4H, s), 3.70 (4H, s), 3.90 (4H, s), 6.52 (2H, s), 6.73-6.74 (2H, d, J = 1.5 Hz), 7.12-7.14 (2H, m), 7.19-7.20 (2H, d, J = 7.5 Hz), 7.55-7.59 (2H, m), 8.46 (2H, d, J = 4.0 Hz), 10.09 (1H, s). ESI-TOF-MS m/z calcd for $C_{36}H_{40}N_5O_5$ $[M+H]^+$ = 622.3027, observed 622.3277.

Synthesis of **9**

To an ice-cooled solution of crude **9-3** (35 mg) in dry CH_2Cl_2 (2 mL) was added dropwise TFA

(2 mL), and the mixture was stirred for 30 min at rt. After removal of the solvent in vacuo, the mixture was purified by reverse-phase HPLC (YMC-Triart C18, 250×10 mm I.D., mobile phase: CH_3CN (0.1% TFA) / H_2O (0.1% TFA) = 20/80 → 60/40, linear gradient over 40 min) to give **9** (12.3 mg, 51% from **9-2**) as an orange solid. The purity of **9** was confirmed to be > 95% by HPLC analysis. ^{1}H-NMR (500 MHz, CD_3CN): 3.19 (4H, t, J = 4.7 Hz), 3.46 (4H, s), 3.93 (4H, s), 4.26 (4H, brs), 6.79 (2H, d, J = 1.5 Hz), 7.10 (2H, t, J = 6.2 Hz), 7.14 (2H, d, J = 7.9 Hz) 7.20 (2H, d, J = 2.0 Hz), 7.66 (2H, t, J = 6.5 Hz), 8.13 (2H, d, J = 4.5 Hz), 9.26 (1H, s). ^{13}C-NMR (125 MHz, CD_3CN): δ 173.6, 160.0, 157.4, 147.2, 145.8, 142.1, 141.3, 125.9, 125.3, 124.3, 115.7, 103.1, 57.9, 57.5, 52.8, 45.2. ESI-TOF-MS *m/z* calcd for $C_{31}H_{32}N_5O_3$ $[M+H]^+$ = 522.2505, observed 522.2524.

Synthetic procedure of probe **10**

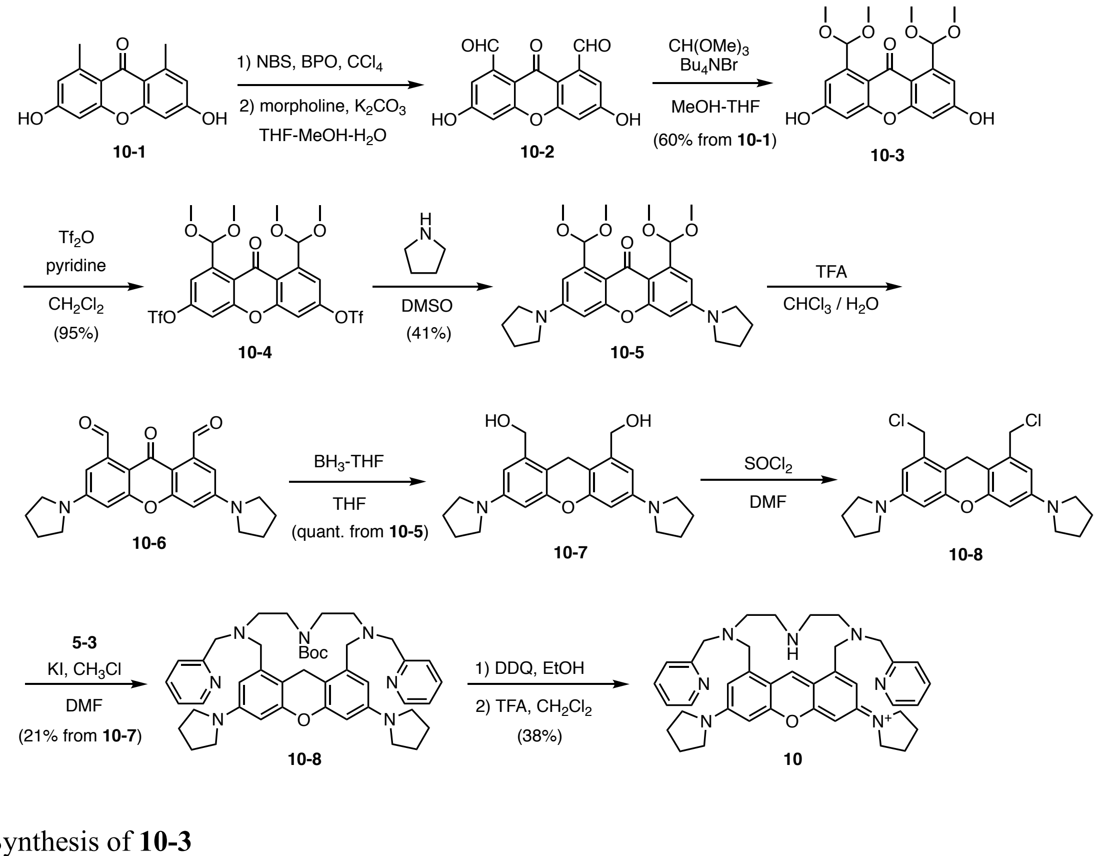

Synthesis of **10-3**

10-1(700 mg, 1.65 mmol) was converted to **10-2** according to the reported method.[S6] The crude **10-2** (1.15 g), trimethyl orthoformate (4.40 mL, 0.365 mmol), and tetrabutylmmonium bromide (206 mg, 0.64 mmol) was dissolved in dry MeOH (20 mL)-dry THF (10 mL). The mixture was stirred for 36 h at 45 °C. After removal of the solvent by evaporation, the residue was purified by column chromatography on SiO_2 ($CHCl_3$: MeOH = 30 : 1) to give a solid. This material was

washed with i-Pr_2O by filtration to give **10-3** as a beige solid (795 mg, 60% yield from **10-1**) ^{1}H-NMR (500 MHz, DMSO-d_6) : 3.38 (12H, s), 6.52 (2H, s), 6.76-6.77 (2H, d, J = 2.5 Hz), 7.08 (2H, d, J = 2.5 Hz). ^{13}C-NMR (125 MHz, DMSO-d_6) : δ 176.7, 161.9, 157.6, 141.9, 112.5, 111.4, 102.0, 100.9, 55.0. ESI-TOF-MS m/z calcd for $C_{19}H_{20}NaO_8$ $[M+Na]^+$ = 399.1056, observed 399.1033.

Synthesis of **10-4**

To an ice-cooled solution of **10-3** (350 mg, 0.93 mmol) in dry CH_2Cl_2 (15 mL) was added dry pyridine (1.13 mL, 14.0 mmol). Trifluoromethansulfonic anhydride (782 μL, 4.65 mmol) was added dropwise and the mixture was stirred for 15 min at 0 °C. After dilution with AcOEt, the organic layer was washed with sat. $NaHCO_3$ aq. and brine followed by drying over Na_2SO_4. After concentration in vacuo, the residues was purified by column chromatography on SiO_2 (hexane : AcOEt = 6 : 1) to give **10-4** (565 mg, 95%) as a colorless solid. ^{1}H-NMR (500 MHz, $CDCl_3$): δ 3.50 (12H, s), 6.59 (2H, s), 7.43-7.44 (2H, d, J = 3 Hz), 7.71 (2H, d, J = 3 Hz). ^{13}C-NMR (125 MHz, $CDCl_3$) : δ 177.0, 156.7, 152.2, 144.4, 119.9, 118.7 (q, $^1J_{C\text{-}F}$ = 319 Hz), 116.6, 111.2, 99.9, 55.2. ESI-TOF-MS m/z calcd for $C_{21}H_{18}F_6NaO_{12}S_2$ $[M+Na]^+$ = 663.0042, observed 663.0126.

Synthesis of **10-5**

In a sealed glass tube, a mixture of **10-4** (540 mg, 0.84 mmol) and pyrrolidine (690 μL, 8.40 mmol) in dry DMSO (3.5 mL) was stirred for 1 h at 85 °C. After dilution with AcOEt, the organic layer was washed with sat. $NaHCO_3$ aq. and brine followed by drying over Na_2SO_4. After removal of the solvent in vacuo, the residue was purified by column chromatography on SiO_2 (hexane : AcOEt =1 : 1) to give **10-5** as a yellow solid (165 mg, 41%).^{1}H-NMR (500 MHz, CD_3OD): 2.07-2.10 (8H, m), 3.41-3.43 (8H, t, J = 6.5 Hz), 6.38-6.39 (2H, d, J = 2.5 Hz), 6.75 (2H, s), 6.95-6.96 (2H, d, J = 2.5 Hz). ^{13}C-NMR (125 MHz, $CDCl_3$) : δ 177.4, 158.3, 150.4, 141.0, 110.2, 107.1, 101.7, 96.9, 55.5, 47.6. ESI-TOF-MS m/z calcd for $C_{27}H_{34}N_2NaO_6$ $[M+Na]^+$ = 505.2315, observed 505.1919.

Synthesis of **10-6**

To an ice-cooled solution of **10-5** (156 mg, 0.32 mmol) in chloroform (4 mL) and water (1 mL) was added dropwise TFA (1 mL), and the mixture was stirred for 15 min at rt. After dilution with sat. $NaHCO_3$ aq., the mixture was extracted with chloroform (x2). The combined organic layers were washed with water and brine followed by drying over Na_2SO_4. The solvent was removed in vacuo to give **10-6** (135 mg) as a yellow solid. ^{1}H-NMR (500 MHz, $CDCl_3$): δ 2.08-2.10 (8H, m), 3.43-3.46 (8H, t, J = 6.5 Hz), 6.49 (2H, d, J = 2.5 Hz), 6.94 (2H, J = 2.5 Hz), 11.12 (2H, s). ^{13}C-NMR (125 MHz, $CDCl_3$) : δ 194.6, 176.5, 157.8, 150.6, 139.6, 110.5, 109.1, 99.9, 47.9, 25.4.

ESI-TOF-MS *m/z* calcd for $C_{23}H_{22}N_2NaO_4$ $[M+Na]^+$ = 413.1477, observed 413.1191.

Synthesis of **10-7**

To an ice-cooled solution of crude **10-6** (135 mg) in dry THF (12 mL) was added dropwise borane-THF complex (0.89 M in THF solution, 2.88 mL, 2.56 mmol), and the mixture was stirred for 30 min at 60°C. After quenching the reaction with H_2O at 0 °C, the resultant mixture was extracted with AcOEt (x2). The combined organic layers were washed with sat. $NaHCO_3$ aq. and brine followed by drying over Na_2SO_4. The solvent was removed in vacuo to give **10-7** (135 mg, quant.) as a light purple solid. ^{1}H-NMR (500 MHz, $CDCl_3$): δ 1.99-2.02 (8H, m), 3.28-3.31 (8H, t, *J* = 6.5 Hz), 3.88 (2H, s), 4.70 (4H, s), 6.19-6.20 (2H, d, *J* = 2 Hz), 6.38 (2H, d, *J* = 2 Hz). ESI-TOF-MS *m/z* calcd for $C_{23}H_{28}N_2NaO_3$ $[M+Na]^+$ = 403.1998, observed 403.1749.

Synthesis of **10-8**

To a solution of **10-7** (65 mg, 0.16 mmol) in dry DMF (2 mL) was added dropwise thionyl chloride (46.5 μL, 0.64 mmol) at rt. The mixture was stirred for 20 min at rt. After dilution with AcOEt, the organic layer with sat. $NaHCO_3$ aq. and brine followed by drying over Na_2SO_4. After removal of the solvent in vacuo, the residue was purified by column chromatography on SiO_2 (hexane : AcOEt = 1 : 1) to give **10-8** (43 mg) as purple solid. This material was used for the next reaction without further purification. The pure sample of **10-8** was obtained by column chromatography on SiO_2 (hexane : CH_2Cl_2 =1 : 1) for structural analysis. ^{1}H-NMR (500 MHz, $CDCl_3$): δ 2.00-2.25 (8H, m), 3.28-3.30 (8H, t, *J* = 6.5 Hz), 4.03 (2H, s), 4.62 (4H, s), 6.23 (2H, brs), 6.34 (2H, brs). ^{13}C-NMR (125 MHz, $CDCl_3$) : δ 152.5, 147.4, 136.2, 108.4, 105.9, 99.6, 47.7, 44.7, 25.4, 20.2. ESI-TOF-MS *m/z* calcd for $C_{23}H_{27}Cl_2N_2O$ $[M+H]^+$ = 417.1500, observed = 417.1529.

Synthesis of **10-9**

A solution of crude **10-8** (43 mg), **5-3** (50 mg, 0.13 mmol), potassium iodide (17 mg, 0.10 mmol), and K_2CO_3 (42 mg, 0.30 mmol) in dry DMF (6 mL) was stirred for 8 h at rt. After dilution with AcOEt, the organic layer was washed with sat. $NaHCO_3$ aq. (x2) and brine followed by drying over Na_2SO_4. The solvent was removed in vacuo and the residue was purified by column chromatography on SiO_2 ($CHCl_3$: MeOH : NH_3 aq. = 80 : 10 : 0.3) to give **10-9** (24 mg, 21%) as a red viscous oil. ^{1}H-NMR (500 MHz, CD_3OD): 1.21 (9H, s), 2.00-2.05 (10H, m), 2.68-2.73 (4H, m), 3.21-3.24 (8H, m),3.35-3.40 (4H, m), 3.63-3.73(8H, m), 4.18-4.24 (2H, m), 6.144 (4H, d, *J* = 2.5 Hz), 7.20 (2H, t, *J* = 6.5 Hz), 7.24 (2H, d, *J* = 8.0 Hz), 7.58-7.62 (2H, m), 8.35 (2H, d, *J* = 5.0 Hz). ESI-TOF-MS *m/z* calcd for $C_{44}H_{56}N_7O_3$ $[M+H]^+$ = 730.4439, observed 730.4406.

Synthesis of **10**

A solution of **10-2** (24.7 mg, 34 μmol) and 2,3-dichloro-5,6-dicyano-*p*-benzoquinone (15 mg, 66 μmol) in dry EtOH (2 mL) was stirred for 1 h at rt. After removal of the solvent in vacuo, the residue was dissolved in dry CH_2Cl_2 (2 mL). TFA (2 mL) was added at 0 °C, and the mixture was stirred for 30 min at rt. After removal of the solvent in vacuo, the residue was purified by reverse-phase HPLC (YMC-Triart C18, 250×10 mm I.D., mobile phase: CH_3CN (0.1% TFA) / H_2O (0.1% TFA) = 20/80 → 60/40, linear gradient over 40 min) to give **10** (8.2 mg, 38%) as a red oil. The purity of **10** was confirmed to be > 95% by HPLC analysis. ^{1}H-NMR (500 MHz, CD_3CN): 1.98-2.06 (8H, m), 3.05-3.70 (20H, m), 3.98 (4H, s), 6.39 (2H, d, J = 2.0 Hz), 7.02 (4H, d, J = 2.0 Hz), 7.10 (2H, d, J = 7.5 Hz), 7.59 (2H, t, J = 7.5 Hz), 8.33 (2H, d, J = 4.0 Hz), 8.92 (1H, d, J = 8.5 Hz). ^{13}C-NMR (125 MHz, CD_3CN): δ 158.9, 158.6, 155.1, 148.4, 142.1, 141.1, 139.3, 125.0, 123.5, 119.5, 113.6, 96.8, 58.1, 57.7, 52.8, 49.8, 45.2, 25.8. ESI-TOF-MS *m/z* calcd for $C_{39}H_{46}N_7O$ $[M]^+$ = 628.3758, observed 628.3759.

Synthetic procedure of probe **11**

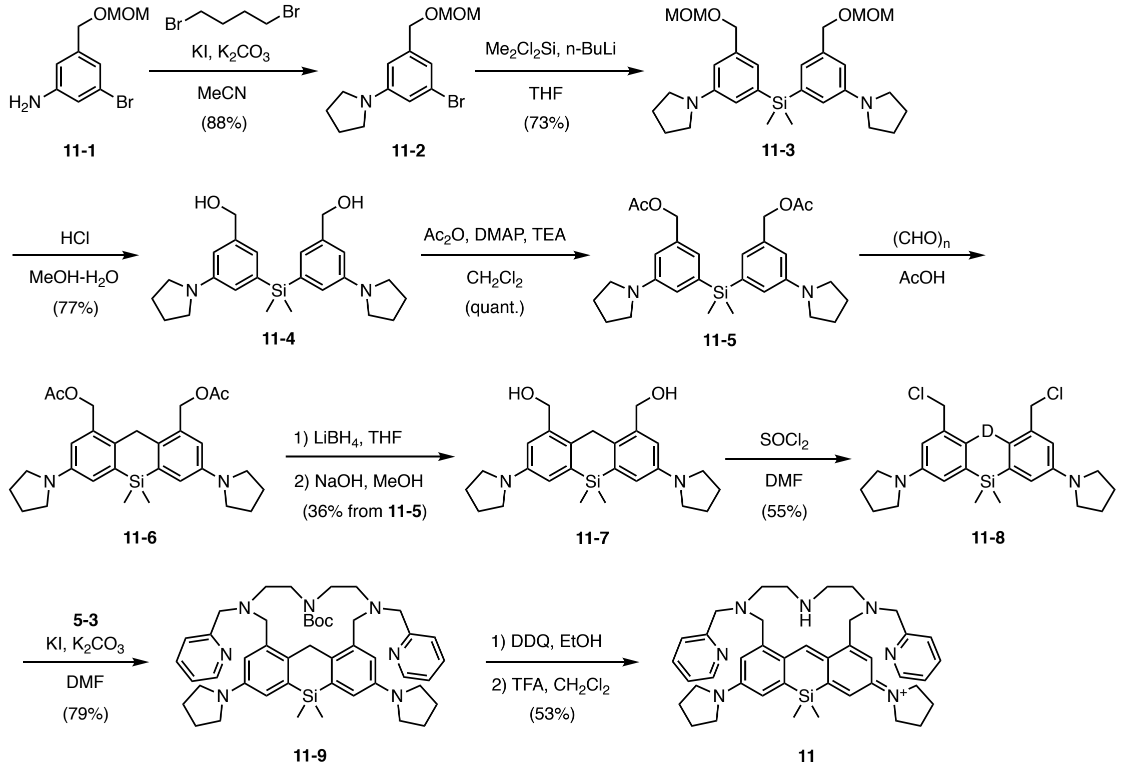

Synthesis **11-2**

A solution of **11-1**[S7] (833 mg, 3.40 mmol), 1,4-dibromobutane (950 mg, 4.40 mmol), K_2CO_3 (1.4 g, 10.2 mmol) and potassium iodide (1.5 g, 8.8 mmol) in dry CH_3CN (24 mL) was refluxed for 35 h with stirring. After removal of K_2CO_3 by filtration, the solution was diluted with AcOEt. The

organic layer was washed with sat. $NaHCO_3$ aq. and brine followed by drying over Na_2SO_4. After removal of the solvent in vacuo, the residue was purified by column chromatography on SiO_2 (hexane : AcOEt = 10 : 1) to give **11-2** (891 mg, 88%) as a colorless oil. ^{1}H-NMR (400 MHz, $CDCl_3$) : δ 1.98-2.01 (4H, m), 3.25-3.28 (4H, m), 3.41 (3H, s), 4.50 (2H, s), 4.69 (2H, s), 6.44 (1H, s), 6.60 (1H, s), 6.77 (1H, s). ^{13}C-NMR (125 MHz, $CDCl_3$): δ 148.9, 140.4, 123.3, 117.3, 113.5, 109.5, 95.6, 124.6, 68.8, 55.4, 47.6, 25.4. ESI-TOF-MS *m/z* calcd for $C_{13}H_{19}BrNO_2$ $[M+H]^+$ = 300.0599, observed 300.0611.

Synthesis of **11-3**

To a cooled (-78 °C) solution of **11-2** (891 mg, 3.0 mmol) in dry THF (9 mL) was added 1.6 M n-butyllithium solution in hexane (2.2 ml, 3.4 mmol). After stirring for 20 min at -78°C, dichlorodimethylsilane (291 µL, 3.0 mmol) was added dropwise and the mixture was stirred for 10 min at -78°C. The mixture was slowly warmed to rt and further stirred for 5 min at rt. After quenching the reaction with H_2O, the resultant mixture was diluted with AcOEt. The organic layer was washed with sat. $NaHCO_3$ aq. and brine followed by drying over N_2SO_4. After removal of the solvent in vacuo, the residue was purified by column chromatography on SiO_2 (hexane : AcOEt = 6 : 1) to give **11-3** (543 mg, 73%) as a colorless oil. ^{1}H-NMR (400 MHz, $CDCl_3$) : δ 0.51 (6H, s), 1.98 (8H, m), 3.27 (8H, m), 3.40 (6H, s), 4.55 (4H, s), 4.69 (4H, s), 6.58 (2H, s), 6.68 (2H, s), 6.82 (2H, s). ESI-TOF-MS *m/z* calcd for $C_{28}H_{43}N_2O_4Si$ $[M+H]^+$ = 499.2992, observed 499.3003.

Synthesis of **11-4**

A solution of **11-3** (543 mg, 2.20 mmol) in MeOH (5 mL)-5 N HCl aq. (5 mL) was stirred for 1 h at 50°C. After dilution with sat. $NaHCO_3$ aq., the resultant mixture was extracted with AcOEt. The organic layer was washed with $NaHCO_3$ aq. and brine followed by drying over N_2SO_4. The solvent was removed in vacuo to give **11-4** (345 mg, 77%) as a yellow solid. ^{1}H-NMR (400 MHz, $CDCl_3$) : δ 0.53 (6H, s), 1.97-2.00 (8H, m), 3.27-3.30 (8H, t, J = 6.6 Hz), 4.62 (4H, s), 6.60 (2H, s), 6.69 (2H, s), 6.81 (2H, s). ESI-TOF-MS *m/z* calcd for $C_{24}H_{35}N_2O_2Si$ $[M+H]^+$ = 411.2468, observed 411.2488.

Synthesis of **11-5**

To a solution of **11-4** (345 mg, 0.84 mmol), triethylamine (468 µL, 3.4 mmol) and 4-dimethylamino pyridine (34 mg, 0.28 mmol) in dry CH_2Cl_2 (10 mL) was added acetic anhydride (238 µL, 2.5 mmol), and the mixture was stirred for 3h at rt. After dilution with dichloromethane, the organic layer was washed with sat. $NaHCO_3$ aq. and brine followed by drying over N_2SO_4. The solvent was removed by evaporation, and the residue was purified by column

chromatography on SiO_2 (hexane : ethyl acetate = 4 : 1) to give **11-5** (415 mg, quant.) as a colorless oil. ^{1}H-NMR (400 MHz, $CDCl_3$) : δ 0.52 (6H, s), 1.97-2.00 (8H, m), 2.01 (6H, s), 3.26-3.29 (8H, t, J = 6.6 Hz), 5.05 (4H, s), 6.55 (2H, s), 6.71 (2H, s), 6.81 (2H, s). ^{13}C-NMR (125 MHz, $CDCl_3$) : δ 170.5, 147.2, 139.0, 135.7, 120.9, 117.0, 112.1, 66.8, 47.3, 25.2, 20.7, -2.4. ESI-TOF-MS *m/z* calcd for $C_{28}H_{39}N_2O_4Si$ $[M+H]^+$ = 495.2679, observed 495.2695.

Synthesis of **11-6**

To the solution of **11-5** (415 mg, 0.84 mmol) in AcOH (5 mL) was added paraformaldehyde (37% solution in H_2O, 366 μL, 4.50 mmol) and the mixture was stirred for 1 h at 70 °C. After dilution with sat. $NaHCO_3$ aq., the mixture was extracted with chloroform (x2). The combined organic layers were washed with sat. $NaHCO_3$ aq. and brine followed by drying over N_2SO_4. The solvent was removed in vacuo to give crude **11-6** (401 mg) as a blue solid. ^{1}H-NMR (400 MHz, $CDCl_3$) : δ 0.52 (6H, s), 1.96-2.02 (6H, m), 2.19 (6H, s), 3.26 (8H, t, J = 7.5 Hz), 5.03 (4H, s), 6.56 (2H, s), 6.61 (2H, s), 6.81 (2H, s). ESI-TOF-MS *m/z* calcd for $C_{28}H_{39}N_2O_4Si$ $[M+H]^+$ = 507.2679, observed 507.2691.

Synthesis of **11-7**

To an ice-cooled solution of lithium borohydride (33.9 mg, 1.60 mmol) in dry THF (6 mL) was slowly added the crude **11-6** (401 mg, 0.79 mmol) dissolved in dry THF (12 mL). The mixture was stirred for 30 min at rt. 1N NaOH aq. (4 mL) and MeOH (4 mL) was added at 0 °C, and the mixture was further stirred for 20 min at rt. After dilution with sat $NaHCO_3$ aq., the resultant mixture was extracted with ethyl acetate (x2). The combined organic layers were washed with sat. $NaHCO_3$ aq. and brine followed by drying over N_2SO_4. After removal of the solvent in vacuo, residue was purified by column chromatography on SiO_2 (hexane : AcOEt = 1 : 1) to give **11-7** (128 mg, 36% from **11-5**) as a colorless solid. ^{1}H-NMR (400 MHz, $CDCl_3$) : δ 0.47 (6H, s), 1.98-2.01 (8H, m), 3.28-3.32 (8H, t, J = 6.8 Hz), 4.10 (2H, s), 4.81 (4H, s), 6.53-6.54 (2H, d, J = 2.8 Hz), 6.76-6.77 (2H, d, J = 2.8 Hz). ^{13}C-NMR (125 MHz, $CDCl_3$) : δ 145.6, 137.8, 137.4, 132.2, 64.2, 47.6, 28.5, 25.1, -3.1. ESI-TOF-MS *m/z* calcd for $C_{25}H_{35}N_2O_2Si$ $[M+H]^+$ = 423.2468, observed 423.2472.

Synthesis of **11-8**

To a solution of **11-7** (46 mg, 0.11 mmol) in dry DMF (2 mL) was added dropwise thionyl chloride (40 μL, 0.55 mmol) at rt. The mixture was stirred for 20 min at rt. After dilution with AcOEt, the organic layer with sat. $NaHCO_3$ aq. and brine followed by drying over Na_2SO_4. After removal of the solvent in vacuo, the residue was purified by column chromatography on SiO_2 (hexane : AcOEt = 10 : 1) to give **11-8** (28 mg, 55%) as a purple solid. ^{1}H-NMR (400 MHz, $CDCl_3$) :

δ0.45 (6H, s), 2.01 (8H, m), 3.31 (8H, m), 4.16 (2H, s), 4.82 (4H, s), 6.56 (2H, s), 6.77 (2H, s). ^{13}C-NMR (125 MHz, $CDCl_3$) : δ 145.8, 137.9, 134.6, 131.4, 116.7, 114.5, 47.7, 46.3, 28.9, 25.4, -2.8. ESI-TOF-MS *m/z* calcd for $C_{25}H_{33}Cl_2N_2O_2Si$ $[M+H]^+$ = 459.1790, observed 459.1801.

Synthesis of **11-9**

A solution of **11-8** (48.5 mg, 0.11 mmol), **5-3** (55 mg, 0.14 mmol), potassium iodide (23.5 mg, 0.14 mmol) and K_2CO_3 (50 mg, 0.36 mmol) in dry DMF (10 mL) was stirred for 8 h at rt. After dilution with sat. $NaHCO_3$ aq., the mixture was extracted with AcOEt (x2). The combined organic layers were washed with sat. $NaHCO_3$ aq. and brine followed by drying over Na_2SO_4. After removal of the solvent in vacuo, the residue was purified by column chromatography on SiO_2 ($CHCl_3$: MeOH : NH_3 aq. = 80 : 1 : 0.1) to give **11-9** (67 mg, 79%) as a blue solid. ^{1}H-NMR (500 MHz, $CDCl_3$) : δ 0.44 (6H, s), 1.23 (9H, s), 1.98-2.01 (8H, m), 2.73 (4H, brs), 3.28-3.31 (8H, t, *J* = 6.3 Hz), 3.62 (4H, brs), 3.71 (4H, s), 3.85 (4H, s), 4.43 (2H, s), 6.50-6.51 (2H, d, *J* = 2.5 Hz), 6.73-6.74 (2H, d, *J* = 2.5 Hz), 7.04-7.07 (2H, q, *J* = 3.0 Hz), 7.26 (2H, m), 7.50 (2H, brs), 8.43-8.44 (2H, d, *J* = 2.5 Hz). ^{13}C-NMR (125 MHz, $CDCl_3$) : δ 170.7, 1159.7, 155.2, 148.5, 145.3, 137.4, 136.0, 135.5, 133.1, 123.0, 121.6, 117.2, 115.3, 80.2, 77.9, 60.5, 53.4, 47.7, 29.6, 28.2, 28.0, 25.4, -2.6. ESI-TOF-MS *m/z* calcd for $C_{46}H_{62}N_7O_2Si$ $[M+H]^+$ = 772.4734, observed 772.4711.

Synthesis of **11**

A solution of **11-9** (16.5 mg, 21 μmol) and 2,3-dichloro-5,6-dicyano-*p*-benzoquinone (11 mg, 48 μmol) in dry EtOH (3 mL) was stirred for 15 min at rt. After removal of the solvent in vacuo, the residue was dissolved in dry CH_2Cl_2 (2 mL). TFA (2 mL) was added at 0 °C, and the mixture was stirred for 30 min at rt. After removal of the solvent in vacuo, the residue was purified by reverse-phase HPLC (YMC-Triart C18, 250×10 mm I.D., mobile phase: CH_3CN (0.1% TFA) / H_2O (0.1% TFA) = 5/95 → 60/40, linear gradient over 40 min) to give **11** (7.5 mg, 53%) as a blue solid. The purity of **11** was confirmed to be > 95% by HPLC analysis. ^{1}H-NMR (500 MHz, CD_3CN) : δ 0.31 (6H, s), 3.19 (4H, s), 3.44 (4H, s), 3.62 (8H, s), 4.00 (4H, s), 4.16 (4H, s), 6.80 (2H, d, *J* = 2.5 Hz), 6.83 (2H, s), 7.22 (2H, d, *J* = 7.5 Hz), 7.29 (2H, t, *J* = 6.5 Hz), 7.74 (2H, t, *J* = 7.5 Hz), 8.38 (2H, d, *J* = 4.5 Hz), 8.49 (1H, s). ^{13}C-NMR (125 MHz, CD_3CN) : δ 158.9, 152.4, 151.6, 149.8, 149.1, 147.9, 140.0, 127.1, 124.9, 123.8, 122.4, 121.2, 121.0, 59.5, 57.7, 53.4, 49.9, 25.8, -0.8. ESI-TOF-MS *m/z* calcd for $C_{41}H_{53}N_7Si$ $[M+H]^{2+}$ = 335.7061, observed 335.7052.

Synthetic procedure of probe **12**

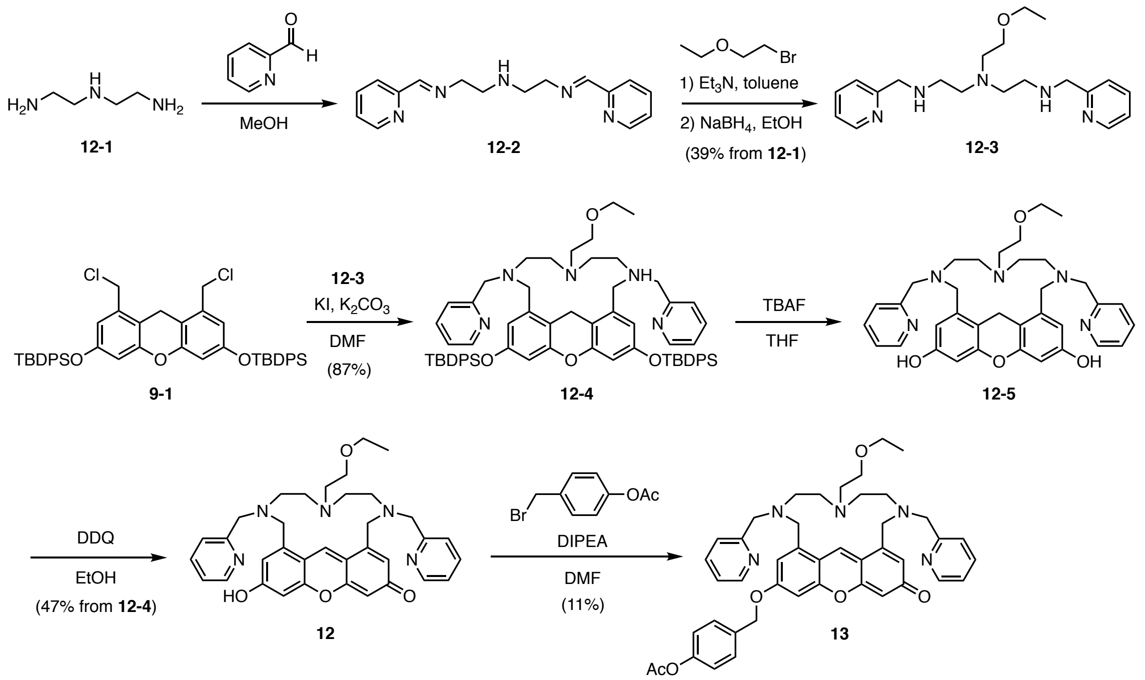

Synthesis of **12-3**

A solution of **12-1** (1.06 mL, 10.0 mmol) and 2-formyl pyridine (2.1 g, 20 mol) in dry ethanol (10 mL) was stirred for 30 min at rt. Sodium sulfate (180 mg) was added and the mixture was further stirred overnight at rt. The solvent was removed by evaporation to give crude **12-2** (2.60 g). This material was used for the next reaction without further purification. ESI-TOF-MS *m/z* calcd for $C_{16}H_{19}N_5Na$ $[M+Na]^+$ = 304.1538, observed 304.1499.

A solution of crude **12-2** (454 mg) 2-bromoethyl ethyl ether (544 μL, 4.82 mmol) and triethylamine (270 μL, 1.94 mmol) in dry toluene (6.0 mL) was stirred for 38 h at 70 °C. After removal of the solvent in vacuo, the residue was diluted with dry EtOH (6 mL). Sodium tetrahydroborate (200 mg, 5.28 mmol) was slowly added and the mixture was stirred for 20 h at 40 °C. After dilution water, the resultant mixture was extracted with chloroform (x2). The combined organic layers was dried over Na_2SO_4. After removal of the solvent in vacuo, the residue was purified by column chromatography on SiO_2 ($CHCl_3$: MeOH : NH_3 aq. = 10 : 1 : 0.1 → 5 : 1 : 0.1) to give **12-3** (112 mg, 39% from **12-1**) as a yellow liquid. ^{1}H-NMR (500 MHz, $CDCl_3$): δ 1.15-1.20 (3H, m), 2.60-2.79 (10H, m), 3.42-3.52 (4H, m), 3.76-3.91 (4H, m), 7.10-7.16 (2H, m), 7.26-7.32 (1H, m), 7.47-7.49 (1H, m), 7.57-7.65 (2H, m), 8.49-8.54 (2H, m). ^{13}C-NMR (125 MHz, $CDCl_3$): δ 159.5, 149.1, 136.3, 122.1, 121.8, 68.8, 66.3, 54.8, 54.3, 53.9, 47.1,

15.0. ESI-TOF-MS *m/z* calcd for $C_{20}H_{32}N_5O$ $[M+H]^+$ = 358.2607, observed 358.2400.

Synthesis of **12-4**

A solution of **9-1**[S6] (60 mg, 76 μmol), **12-3** (36 mg, 0.10 mmol), potassium iodide (13 mg, 78 μmol) and K_2CO_3 (32 mg, 0.23 mmol) in dry DMF (6 mL) was stirred for 7 h at rt. After dilution with sat. $NaHCO_3$ aq., the mixture was extracted with AcOEt (x2). The combined organic layers were washed with sat. $NaHCO_3$ aq. and brine followed by drying over Na_2SO_4. After removal of the solvent in vacuo, the residue was purified by column chromatography on SiO_2 ($CHCl_3$: MeOH : NH_3 aq. = 30 : 1 : 0.1 → 20 : 1 : 0.1) to give **12-4** (71 mg, 87%) as a colorless oil. ^{1}H-NMR (500 MHz, $CDCl_3$): δ 1.09 (18H, s), 2.21 (2H, t, *J* = 7.5 Hz), 2.44 (4H, brs), 2.49 (4H, brs), 2.66-2.75 (3H, m), 3.16 (6H, brs), 3.18 (2H, q, *J* = 9.0 Hz), 3.41-3.50 (4H, m), 3.92 (1H, S), 4.41 (2H, s), 6.23 (2H, s), 6.51 (2H, s), 6.90 (2H, d, *J* = 9.5 Hz), 7.00 (2H, dt, *J* = 1.0 Hz, 7.5 Hz), 7.23-7.25 (2H, m), 7.27-7.34 (18H, m), 7.69 (4H, d, *J* = 2.5 Hz), 7.71 (4H, d, *J* = 2.5 Hz), 8.39 (2H, d, *J* = 2.5 Hz), ESI-TOF-MS *m/z* calcd for $C_{67}H_{78}N_5O_4Si_2$ $[M+H]^+$ = 1072.5592, observed 1072.5635.

Synthesis of **12**

To a solution of **12-4** (24.7 mg, 23 μmol) in dry THF (2 mL) was added tetrabutylammonium fluoride in THF (1 mol/L; 73.7 μL, 73.7 μmol), and the mixture was stirred for 1 h at rt. After removal of the solvent in vacuo, the residue was dissolved in dry EtOH (2 mL). 2,3-Dichloro-5,6-dicyano-*p*-benzoquinone (8.5 mg, 37 μmol) was added and the mixture was stirred for 10 min at rt. After removal of the solvent in vacuo, the residue was purified by column chromatography on SiO_2 ($CHCl_3$: MeOH = 5 : 1 → 7 : 1 → $CHCl_3$: MeOH : NH_3 aq. = 7 : 1 : 0.1) to give **13** (6.4 mg, 47%) as an orange oil. ^{1}H-NMR (500 MHz, $CDCl_3$): δ 0.99 (3H, t, *J* = 7.0 Hz), 2.50 (2H, s), 2.69 (8H, br), 3.16 (3H, br), 3.26 (2H, q, *J* = 7.0 Hz), 3.34 (2H, br), 3.60 (4H, brs), 3.64 (1H, s), 6.51 (2H, s), 6.73 (2H, s), 7.06 (2H, t, *J* = 5.8 Hz), 7.13 (2H, t, *J* = 6.8 Hz), 7.47 (2H, t, *J* = 8.1 Hz), 8.38 (2H, d, *J* = 4.1 Hz), 9.89 (1H, brs). ^{13}C-NMR (125 MHz, $CDCl_3$): δ 159.6, 158.9, 148.5, 141.3, 140.9, 136.2, 128.5, 127.5, 123.5, 121.8, 113.4, 102.8, 67.7, 66.3, 60.0, 57.0, 52.9, 51.9, 51.0, 15.1. ESI-TOF-MS *m/z* calcd for $C_{35}H_{40}N_5O_4$ $[M+H]^+$ = 594.3080, observed 594.3087.

Synthesis of **13**

To a solution of **12** (9.5 mg, 16.0 μmol) and DIPEA (16.8 μL, 96.0 μmol) in dry DMF (1.5 mL) was added (4-bromomethyl)phenylacetate (13.2 mg, 57.6 μmol) and the mixture was stirred for 17 h at rt. After dilution with AcOEt, the organic layer was washed with sat. $NaHCO_3$ aq. and brine followed by drying over Na_2SO_4. After removal of the solvent, the residue was purified by reverse-phase HPLC (YMC-Triart C18, 250×10 mm I.D., mobile phase: CH_3CN (0.1% TFA) /

H_2O (0.1% TFA) = 5/95 → 60/40, linear gradient over 40 min) to give **12** (1.2 mg, 11%) as a yellow solid. The purity of **13** was confirmed to be > 95% by HPLC analysis. ^{1}H-NMR (500 MHz, CD_3CN): δ = 7.70 (2H, dd, *J* = 3.5Hz, 5.0 Hz), 7.60 (2H, dt, *J* = 1.5 Hz, 8.0 Hz), 7.46 (2H, d, *J* = 8.5 Hz), 7.36 (2H, d, *J* = 8.5 Hz), 7.16 (2H, d, *J* = 8.5 Hz), 7.04-7.09 (5H, m), 6.76 (2H, d, *J* = 8.5 Hz), 4.56 (2H, s), 4.22-4.26 (4H, m), 3.52-3.75 (4H, m), 3.07-3.12 (4H, m), 2.24 (s, 3H), 0.89 (3H, t, *J* = 3.5 Hz). ESI-TOF-MS *m/z* calcd for $C_{44}H_{48}N_5O_6$ $[M+H]^+$ = 742.3605, observed 742.3564.

Supplementary References

S1. Ojida, A.; Mito-oka, Y.; Inoue, M.; Hamachi, I. *J. Am. Chem. Soc.* **2002**, *124*, 6256-6258.

S2. Grisenti, A. L.; Smith, M. B.; Fang, L.; Bishop, N.; Wagenknecht, P. S.; *Inorg. Chim. Acta* **2010,** *363*, 157-162.

S3. Zhang, Y.-M.; Chang, D.-C.; Zhang, J.; Liu, Y.-H.; Yu, X.-Q. *Bioorg. Med. Chem.* **2015,** *23*, 5756-5763.

S4. Matouzenko, G. S.; Borshch, S. A.; Jeanneau, E.; Bushuev, M. B.; *Chem. Eur. J.* **2009,** *15*, 1252-1260.

S5. Atkins, R. L.; Bliss, D. E.; *J. Org. Chem.*, **1978,** *43*, 1975-1980.

S6. Takashima, I.; Kanegae, A.; Sugimoto, M.; Ojida, A.; *Inorg. Chem.* **2014,** *53*, 7080-7082.

S7. Reuter, R.; Wegner, H. A.; *Chem. Eur. J.* **2011,** *17*, 2987-2995.

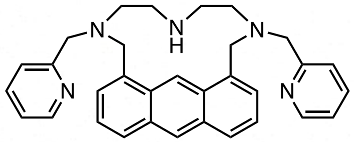

5

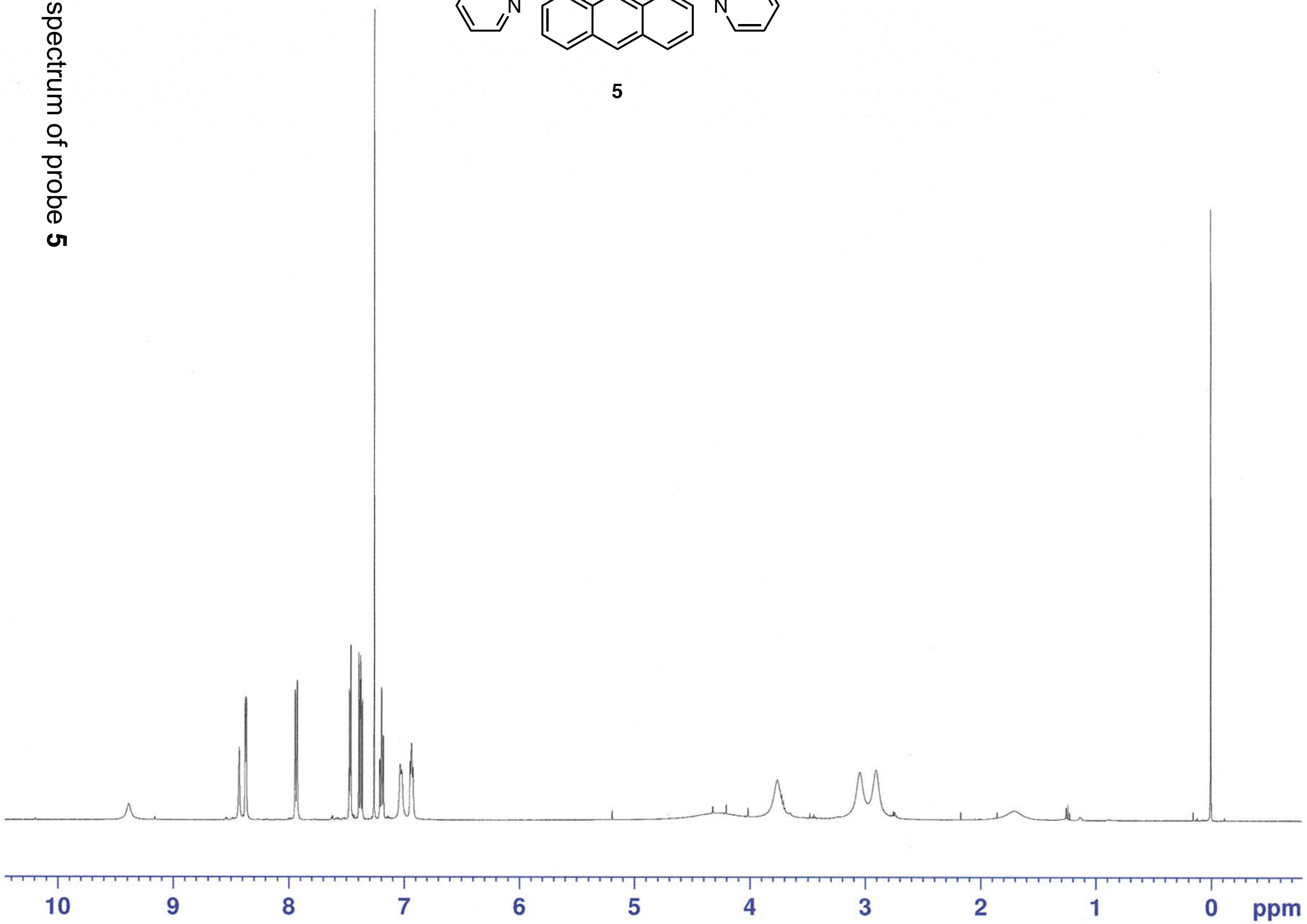

Current Data Parameters
NAME Dec25-2020-anth
EXPNO 10
PROCNO 1

F2 - Acquisition Parameters
Date_ 20201225
Time 11.55 h
INSTRUM spect
PROBHD Z130033_0007 (
PULPROG zg30
TD 65536
SOLVENT CDCl3
NS 16
DS 2
SWH 10000.000 Hz
FIDRES 0.305176 Hz
AQ 3.2767999 sec
RG 31.29
DW 50.000 usec
DE 13.55 usec
TE 300.0 K
D1 1.00000000 sec
TD0 1
SFO1 500.1730885 MHz
NUC1 1H
P0 4.00 usec
P1 12.00 usec
PLW1 13.50000000 W

F2 - Processing parameters
SI 65536
SF 500.1700121 MHz
WDW EM
SSB 0
LB 0.30 Hz
GB 0
PC 1.00

^{1}H-NMR spectrum of probe **5**

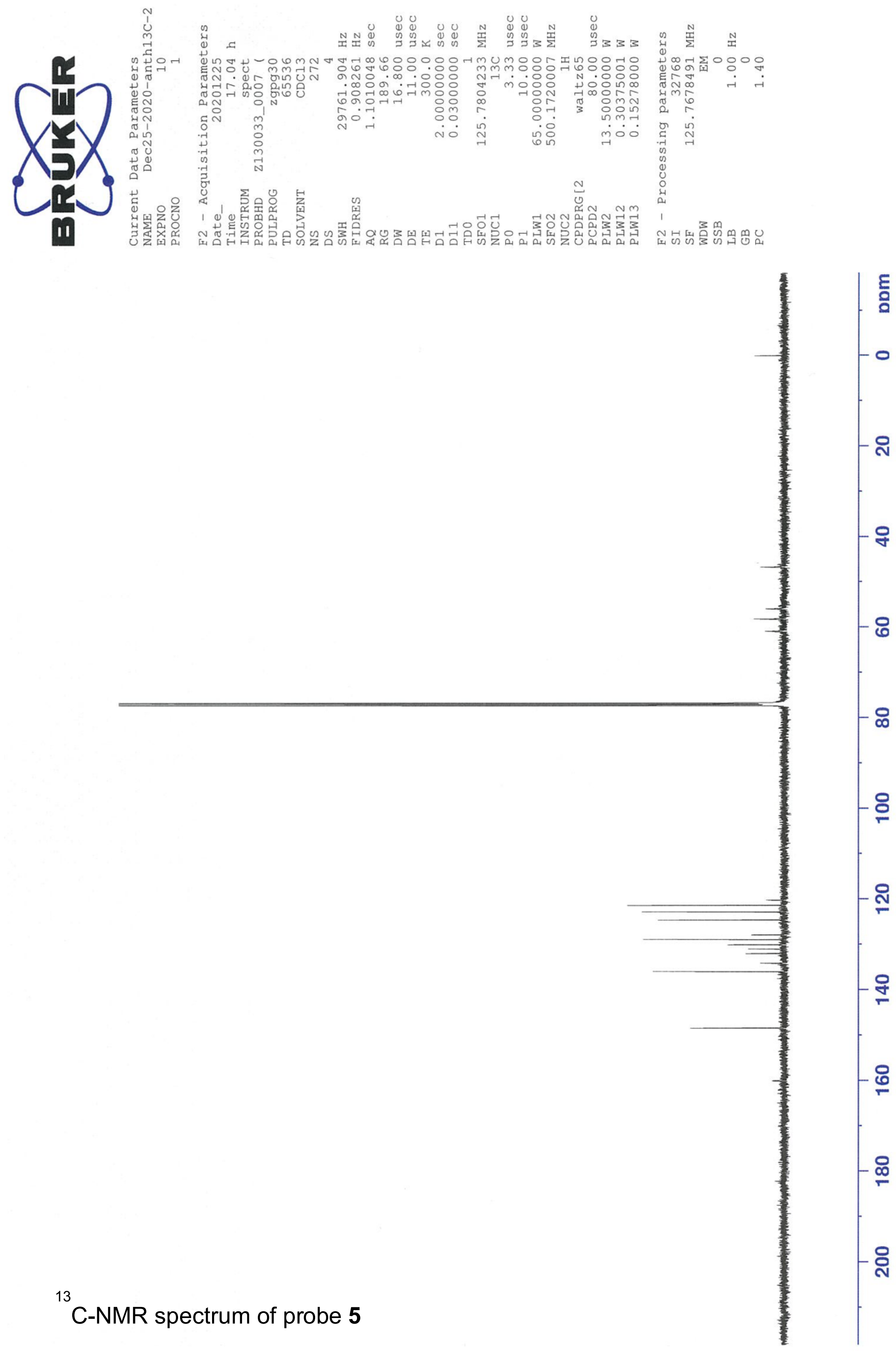

^{13}C-NMR spectrum of probe **5**

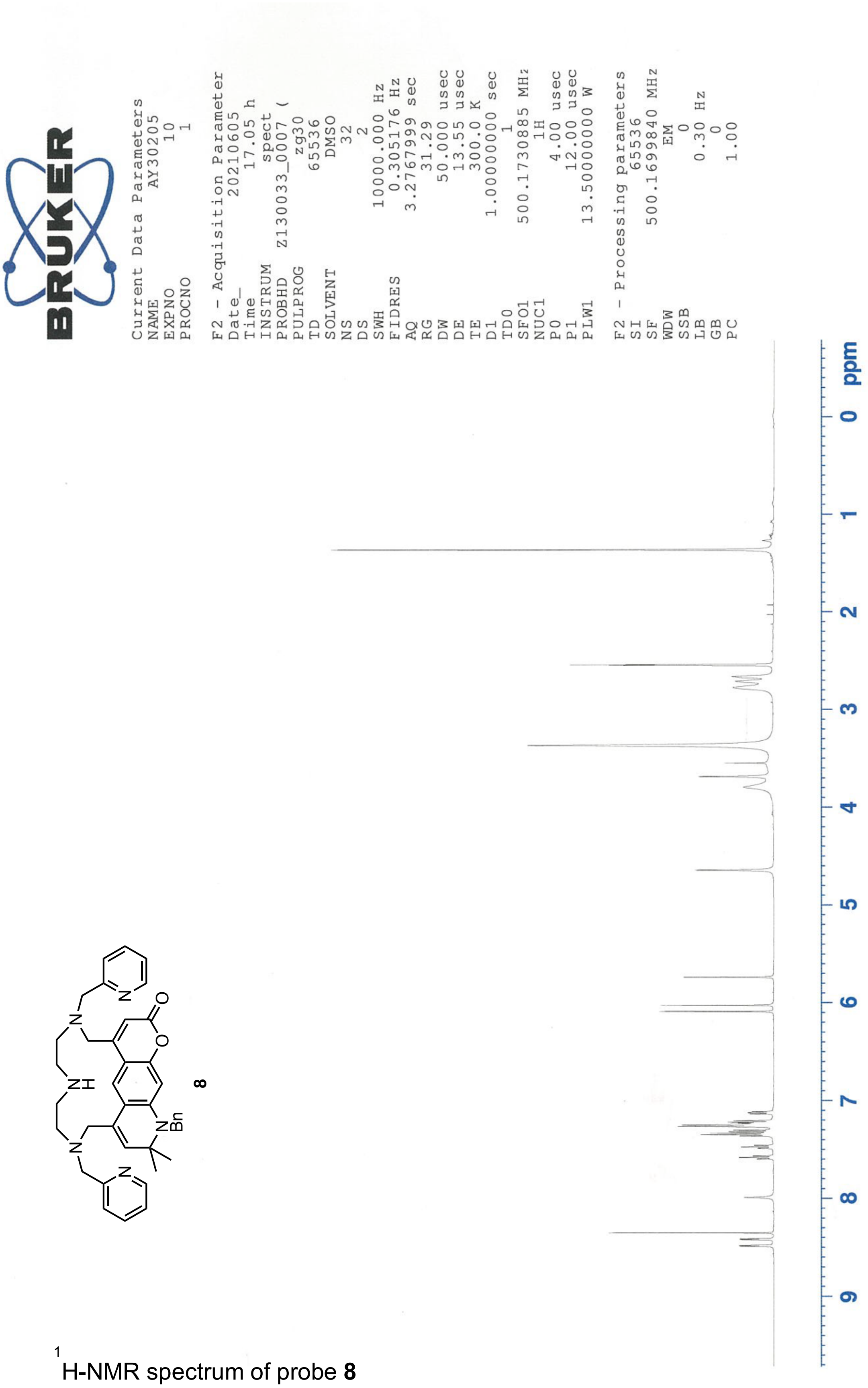

^{1}H-NMR spectrum of probe **8**

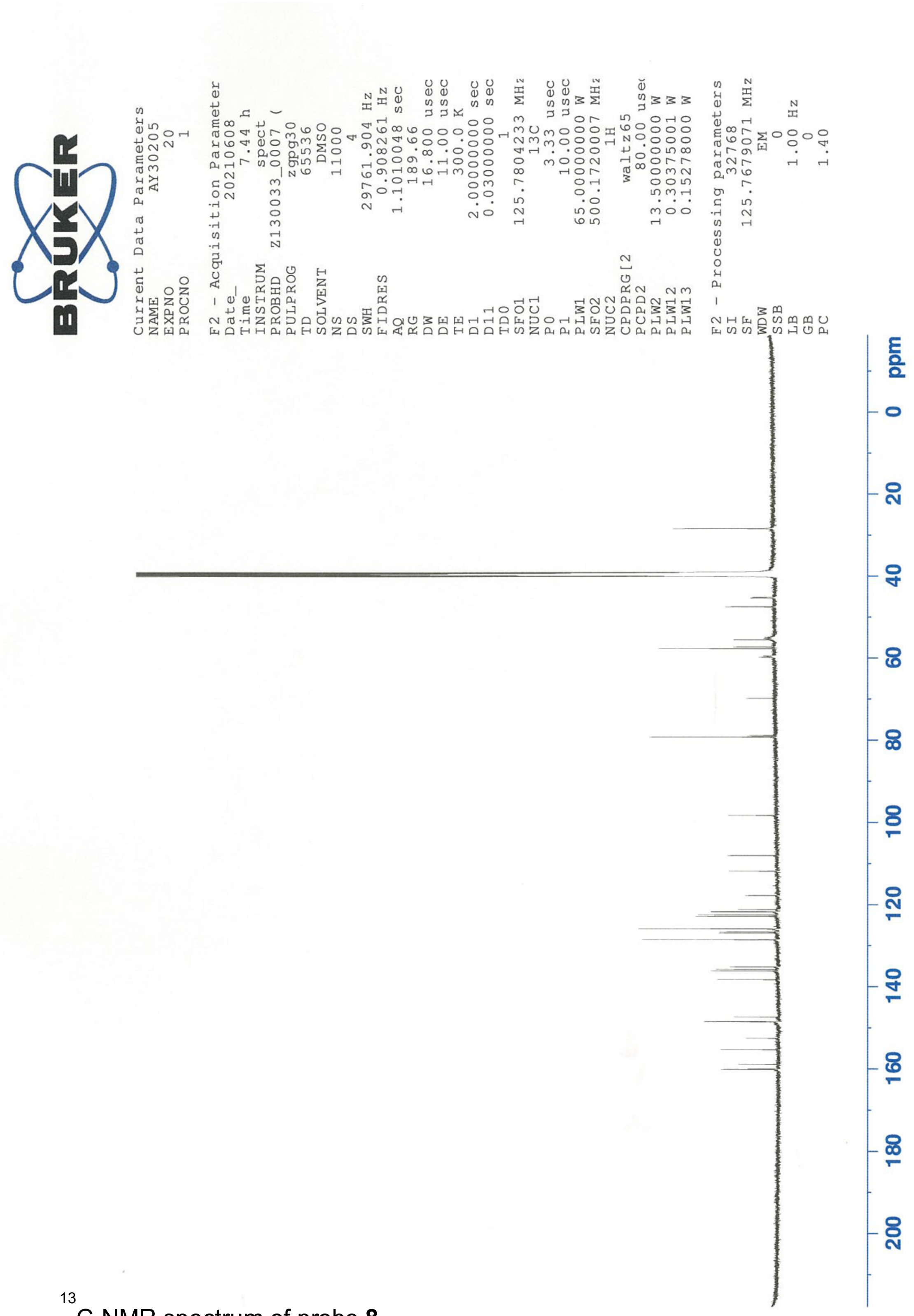

^{13}C-NMR spectrum of probe **8**

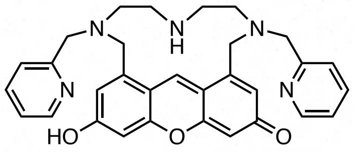

9

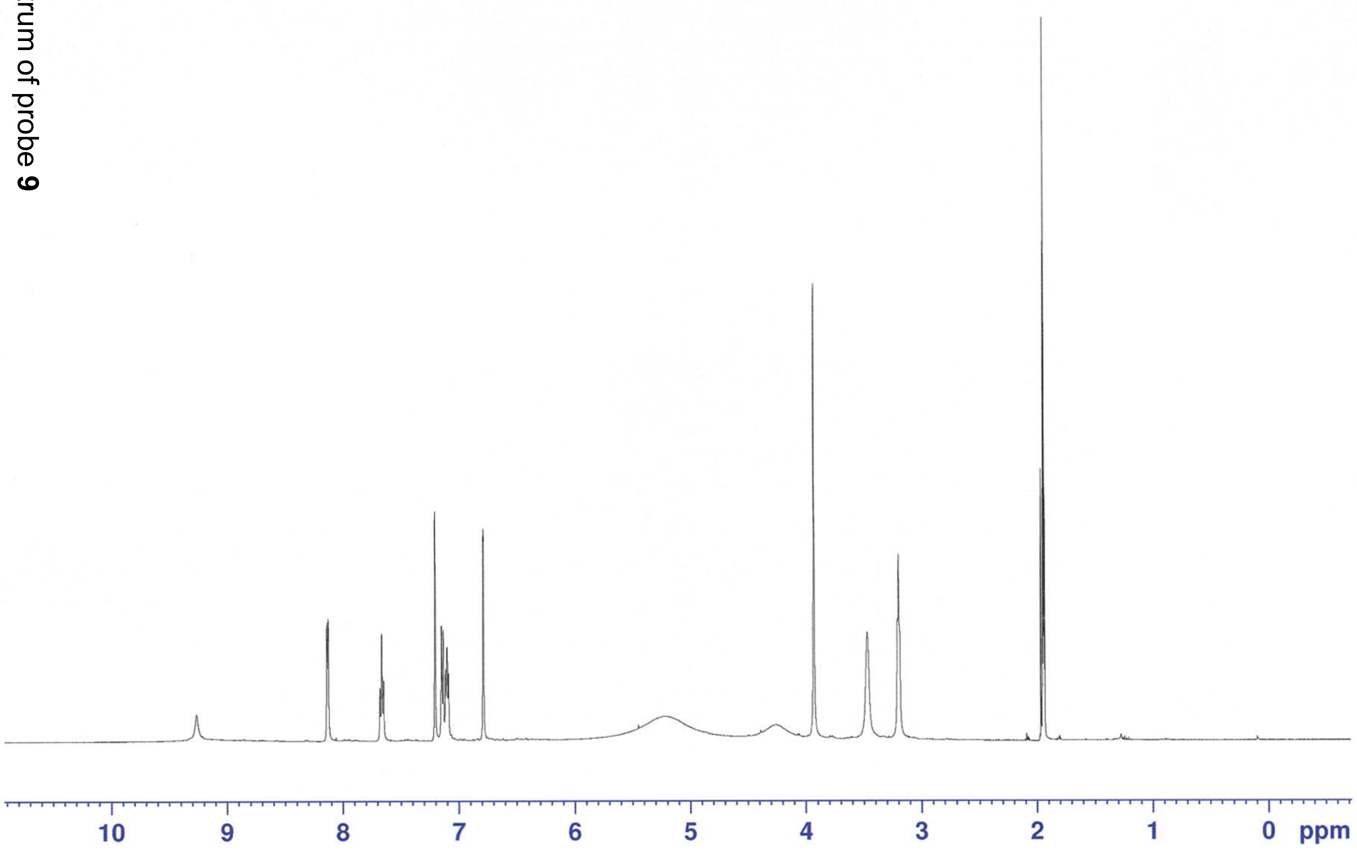


```
Current Data Parameters
NAME                AY31003
EXPNO                    10
PROCNO                    1

F2 - Acquisition Parameters
Date_              20210411
Time                  17.04 h
INSTRUM               spect
PROBHD    Z130033_0007 (
PULPROG                zg30
TD                    65536
SOLVENT               CD3CN
NS                       32
DS                        2
SWH              10000.000 Hz
FIDRES            0.305176 Hz
AQ               3.2767999 sec
RG                    31.29
DW                   50.000 usec
DE                    13.55 usec
TE                    300.0 K
D1              1.00000000 sec
TD0                       1
SFO1           500.1730885 MHz
NUC1                     1H
P0                     4.00 usec
P1                    12.00 usec
PLW1            13.50000000 W

F2 - Processing parameters
SI                    65536
SF             500.1700142 MHz
WDW                      EM
SSB                       0
LB                     0.30 Hz
GB                        0
PC                     1.00
```

^{1}H-NMR spectrum of probe **9**

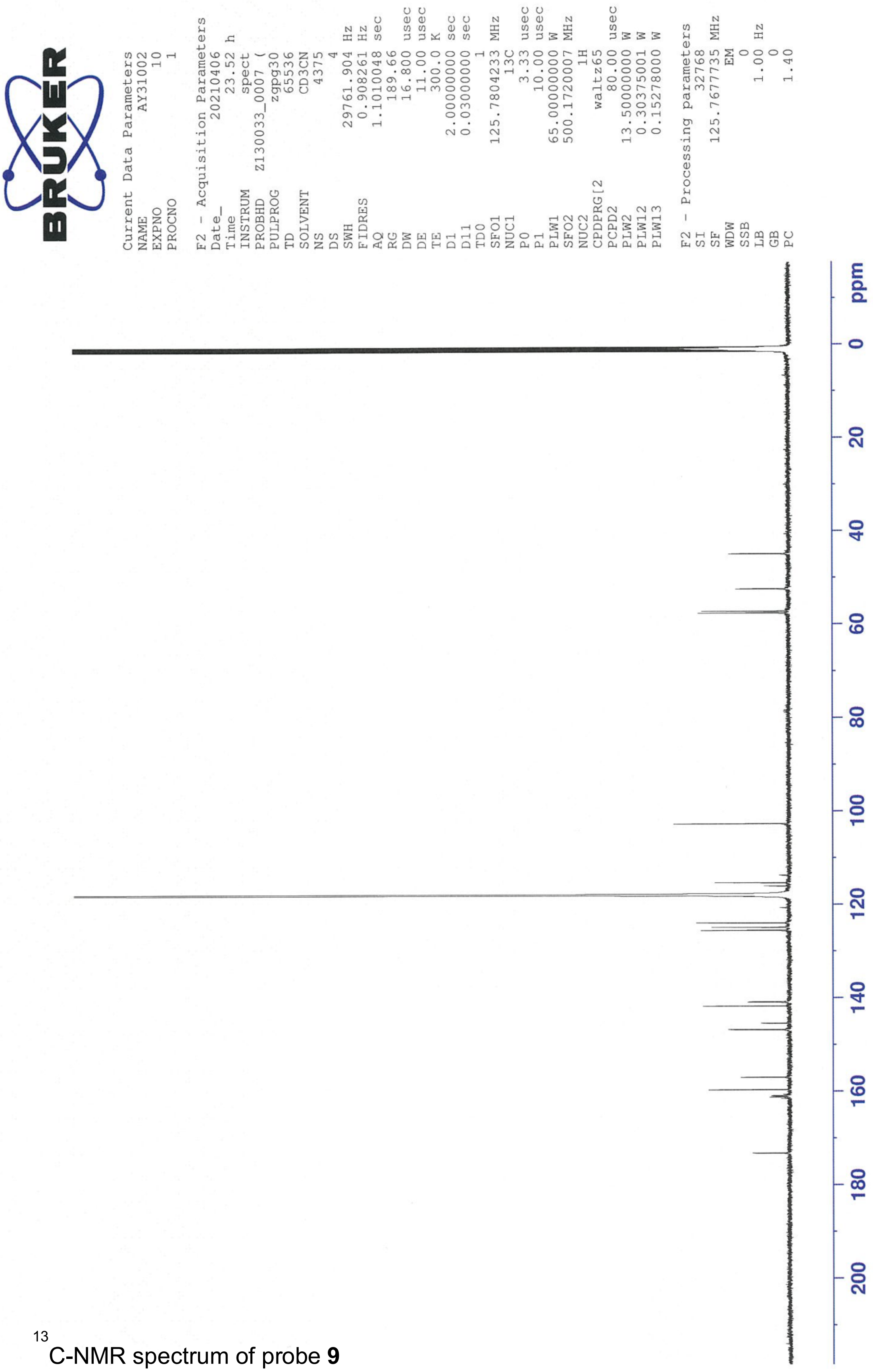

^{13}C-NMR spectrum of probe **9**

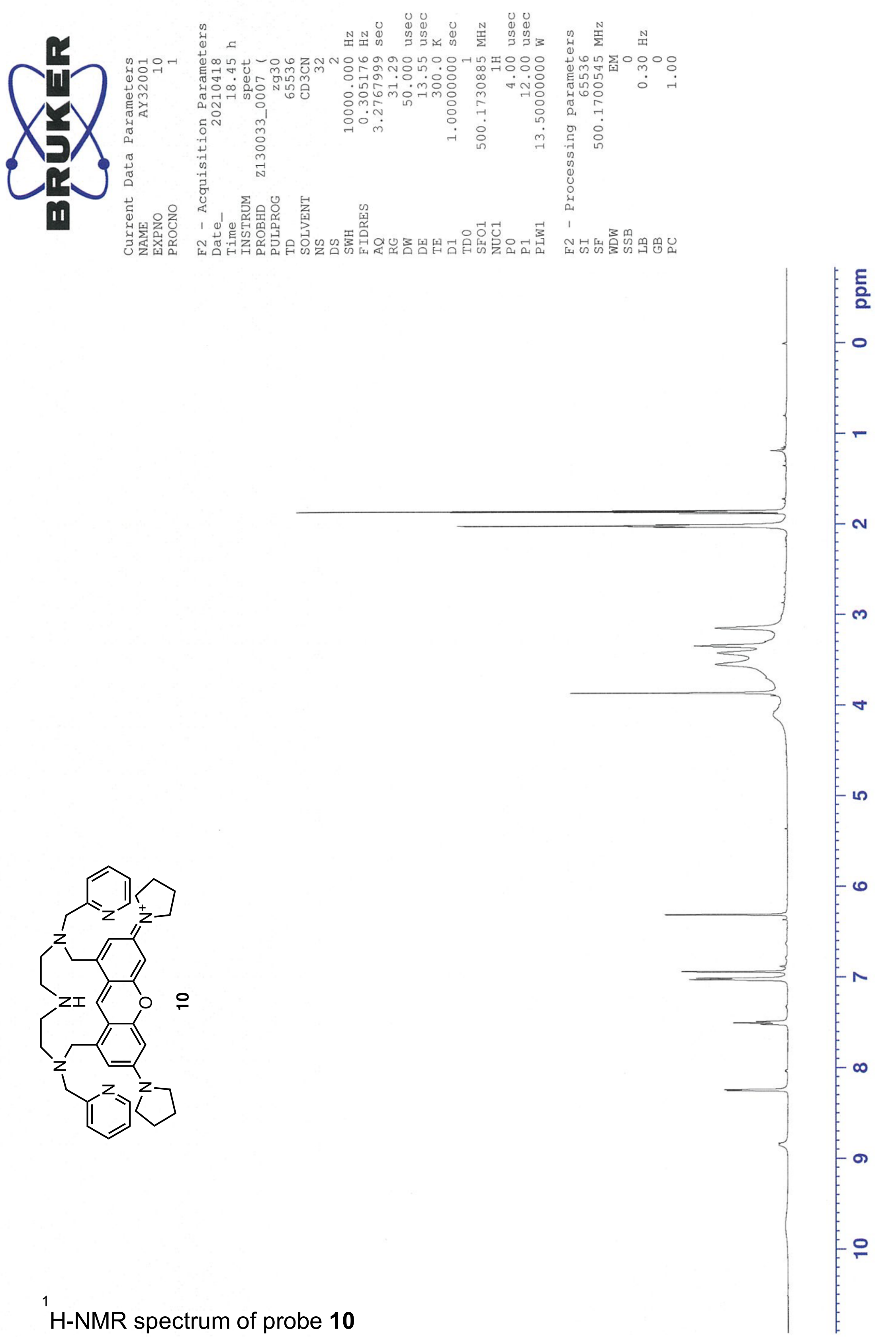

^{1}H-NMR spectrum of probe **10**

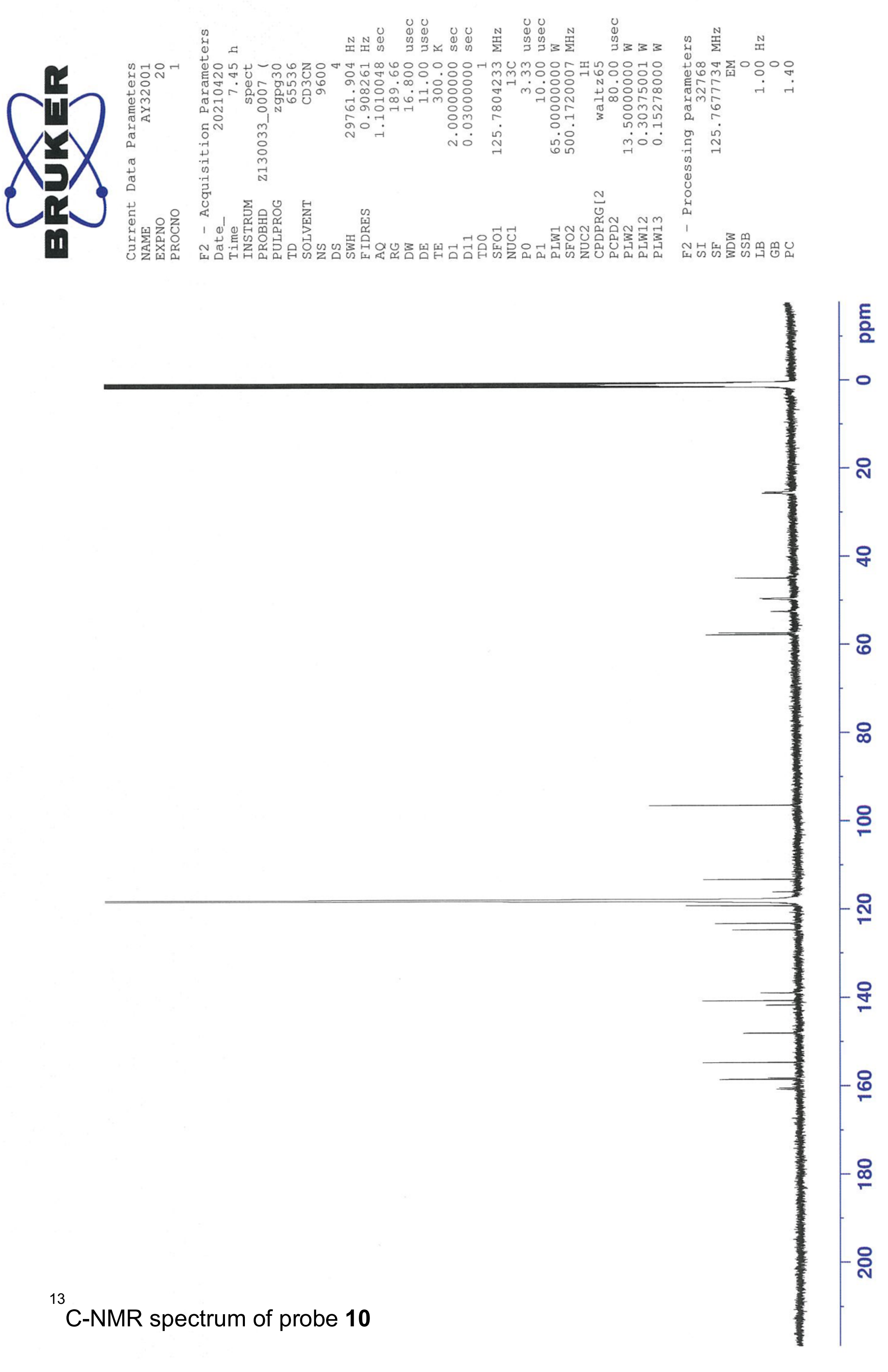

^{13}C-NMR spectrum of probe **10**

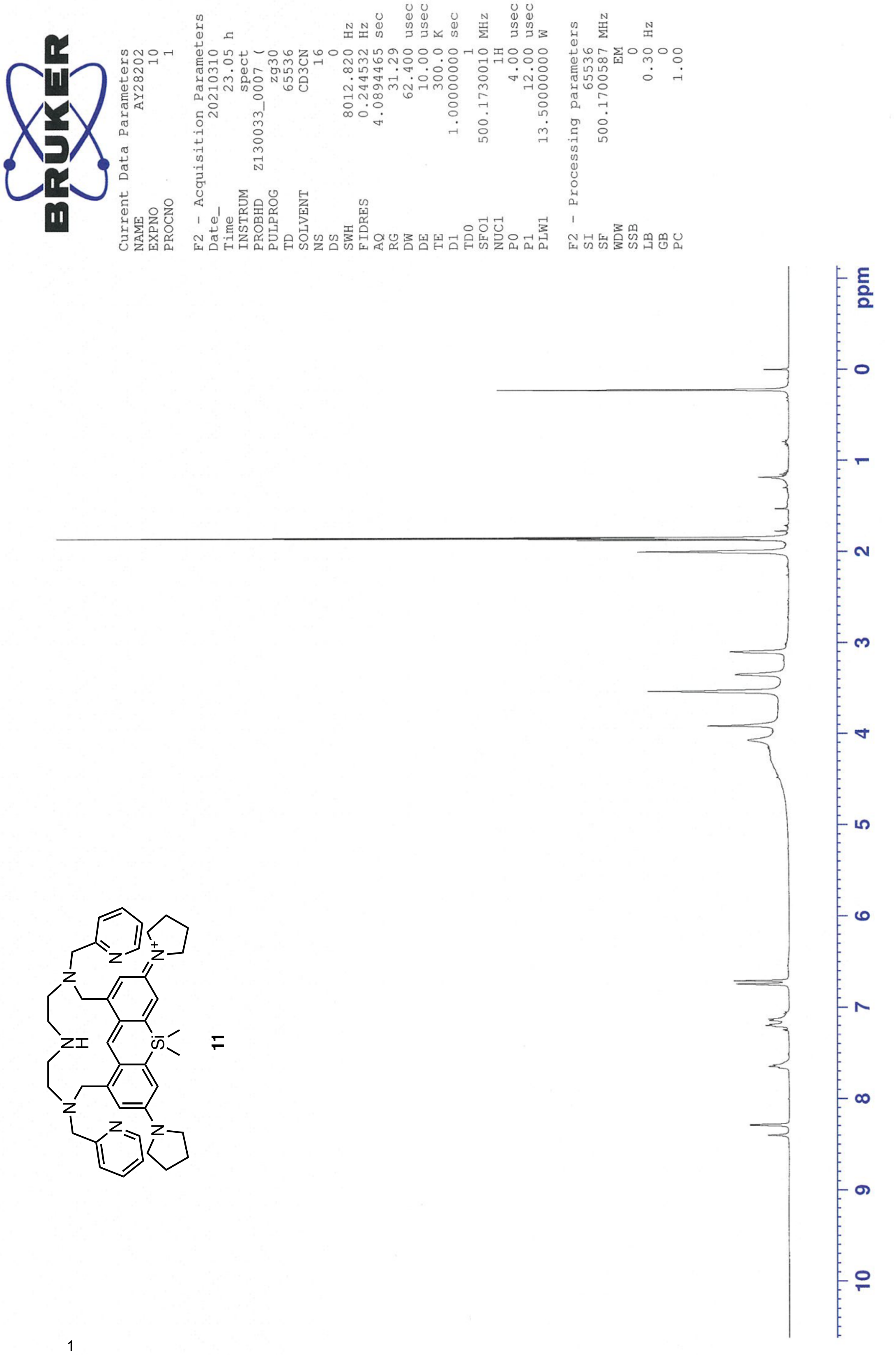

^{1}H-NMR spectrum of probe **11**

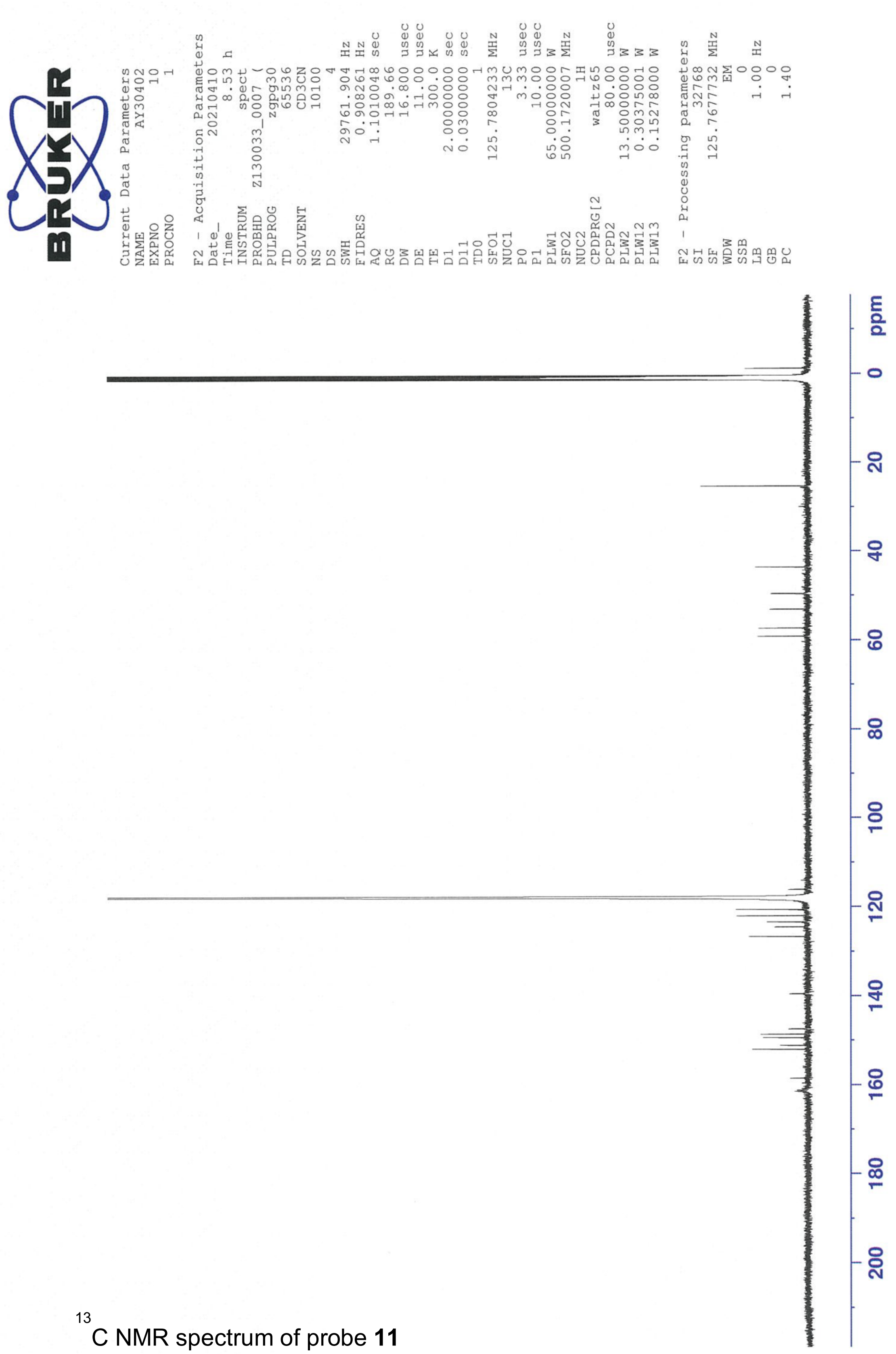

^{13}C NMR spectrum of probe **11**

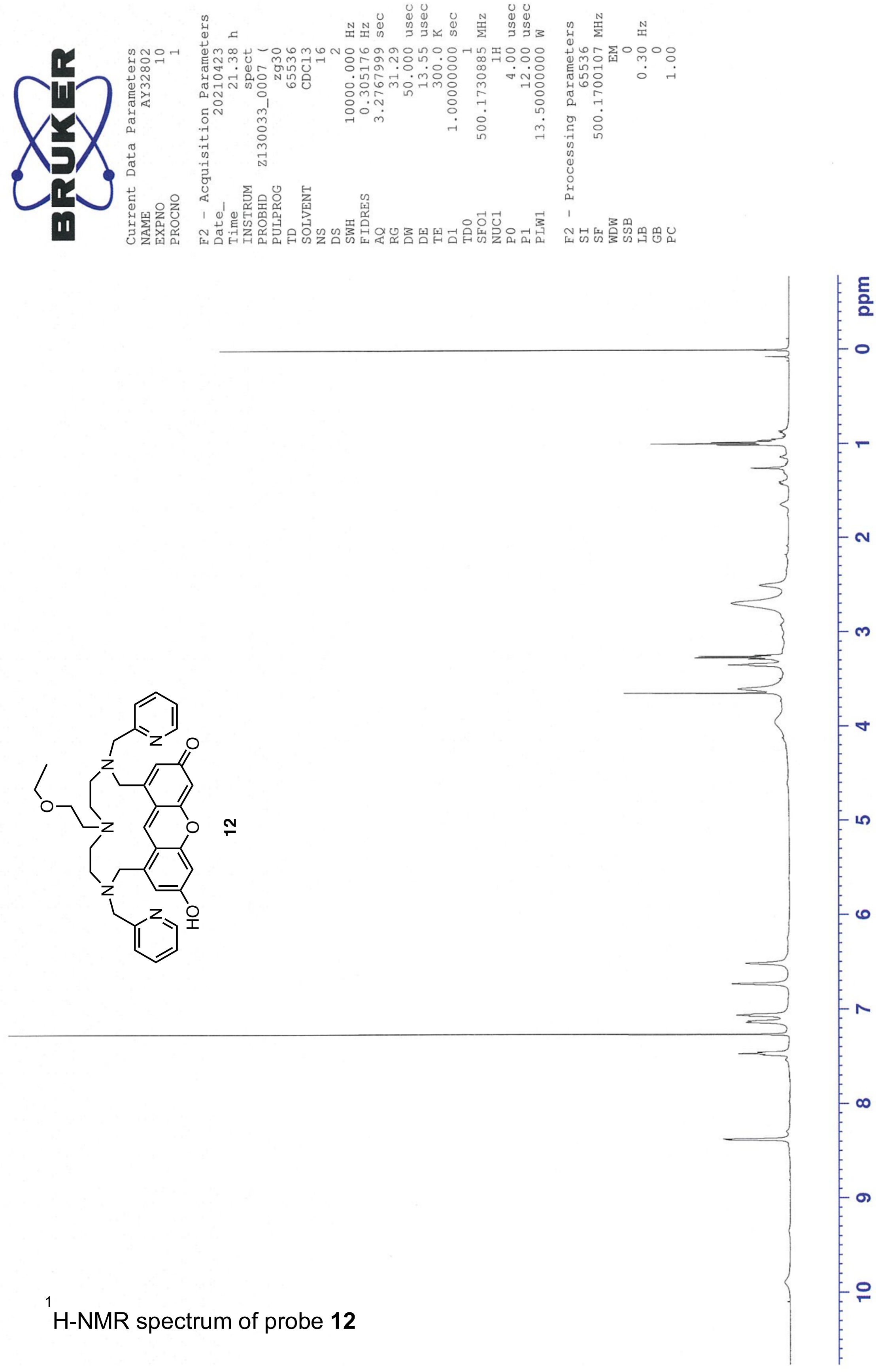

^{1}H-NMR spectrum of probe **12**

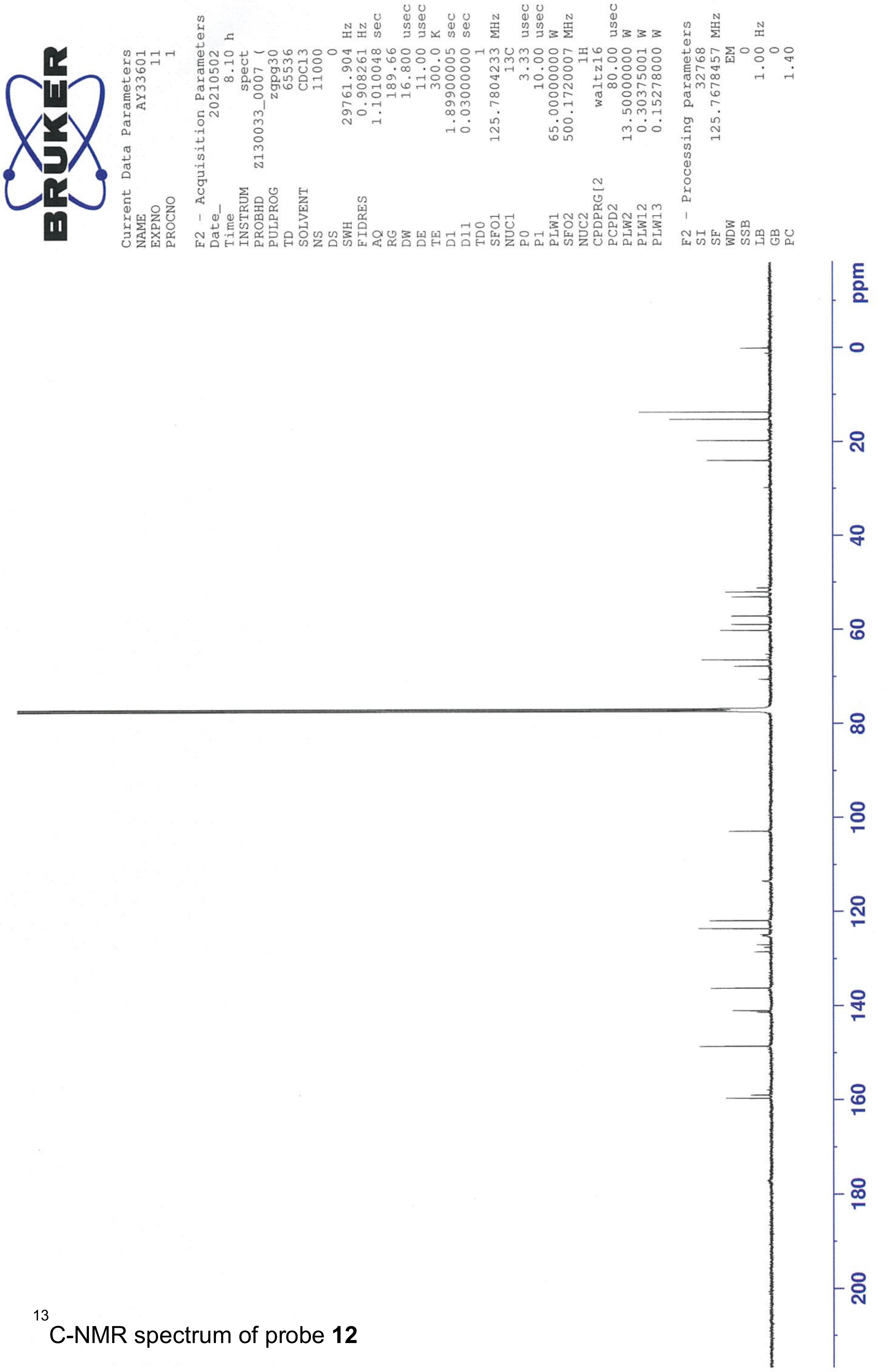

^{13}C-NMR spectrum of probe **12**
