# Supplementary material for: A multicolor and ratiometric fluorescent sensing platform for metal ions based on arene–metal-ion contact
Source: Commun Chem. 2021 Jul 6;4:104. doi: 10.1038/s42004-021-00541-y (PMC9814090; doi:10.1038/s42004-021-00541-y)
Supplement: Supplementary file 7 — Description of Additional Supplementary Files. [file 42004_2021_541_MOESM7_ESM.pdf]

Description of Additional Supplementary Files

File Name: Supplementary Data 1

Description: crystallographic information file (CIF) of compound 5-Zn(II).

File Name: Supplementary Data 2

Description: crystallographic information file (CIF) of compound 5-Cd(II).

File Name: Supplementary Data 3

Description: crystallographic information file (CIF) of compound 5-Ag(I).

File Name: Supplementary Data 4

Description: crystallographic information file (CIF) of compound 5-Cu(II).
